# The Effects of 52 Weeks of Soccer or Resistance Training on Body Composition and Muscle Function in +65-Year-Old Healthy Males – A Randomized Controlled Trial

**DOI:** 10.1371/journal.pone.0148236

**Published:** 2016-02-17

**Authors:** Thomas Rostgaard Andersen, Jakob Friis Schmidt, Mogens Theisen Pedersen, Peter Krustrup, Jens Bangsbo

**Affiliations:** 1 Copenhagen Centre for Team Sport and Health, Department of Nutrition, Exercise and Sports, University of Copenhagen, Copenhagen, Denmark; 2 Department of Cardiology, Gentofte Hospital, Gentofte, Denmark; 3 Sport and Health Sciences, College of Life and Environmental Sciences, University of Exeter, Exeter, United Kingdom; University of Alabama at Birmingham, UNITED STATES

## Abstract

The effects of 52 weeks of soccer or resistance training were investigated in untrained elderly men. The subjects aged 68.1±2.1 yrs were randomised into a soccer (SG; n = 9), a resistance (RG; n = 9) and a control group (CG; n = 8). The subjects in SG and RG, respectively, trained 1.7±0.3 and 1.8±0.3 times weekly on average during the intervention period. Muscle function and body composition were determined before and after 16 and 52 weeks of the intervention period. In SG, BMI was reduced by 1.5% and 3.0% (p<0.05) after 16 and 52 weeks, respectively, unchanged in RG and 2% higher (p<0.05) in CG after 52 weeks of the intervention period. In SG, the response to a glucose tolerance test was 16% lower (p<0.05) after 16 wks, but not after 52 wks, compared to before the intervention period, and unchanged in RG and CG. In SG, superoxide dismutase-2 expression was 59% higher (p<0.05) after 52 wks compared to before the intervention period, and unchanged in RG and CG. In RG, upper body lean mass was 3 and 2% higher (p<0.05) after 16 and 52 wks, respectively, compared to before the intervention period, and unchanged in SG and CG. In RG, Akt-2 expression increased by 28% (p<0.01) and follistatin expression decreased by 38% (p<0.05) during the 52-wk intervention period, and was unchanged in SG and CG. Thus, long-term soccer training reduces BMI and improves anti-oxidative capacity, while long-term resistance training impacts muscle protein enzyme expression and increases lean body mass in elderly men.

**Trial Registration:**

ClinicalTrials.gov: NCT01530035

## Introduction

Aging impacts muscle morphology resulting in a decrease in fibre size and muscle capillarization, changes in fibre-type distribution towards an increased expression of type IIX muscle fibres, and may lead to reductions in mitochondrial content and muscle function [1]. In addition, increased adiposity and oxidative stress are commonly observed in elderly sedentary subjects leading to an increased risk of developing life-style related disease [2]. Exercise training has repeatedly been shown to counteract these adverse effects [3]. Thus, exercise interventions designed to preserve muscle function and/or muscle mass, reduce adiposity and enhance antioxidant capacity may prove vital for an independent lifestyle, and successful, healthy aging.

The physical activity recommendations for aging subjects include a minimum of 150 min of moderate intensity or 60 min of vigorous physical activity per week [4], and resistance training recommendations comprise 10–15 repetitions of 8–10 exercises that involve major muscle groups at moderate to high intensity at least twice per week [4]. Moreover, a combination of resistance and aerobic training seems more efficient than either of the training forms alone [5].

In recent years, the health benefits of recreational soccer have been comprehensively investigated [6;7]. With respect to the aging subject, recreational soccer has been shown to stimulate both the aerobic and anaerobic energy systems, as indicated by high heart rates and elevated blood lactate levels during training for untrained elderly players (+65 yrs) [8]. In elderly men with life-long participation in soccer, cardiac function, exercise performance and body composition were shown to be superior when compared to age-matched untrained males [9], and when untrained, healthy elderly men engage in 16 weeks of soccer training substantial health and performance adaptations have been shown to occur [8]. Therefore, recreational soccer training may serve as an important alternative to training modalities traditionally applied to maintain physical function, health, and longevity in the aging population such as resistance and endurance training [10].

The present study represented an independent part of a comprehensive 52-wks interventional protocol investigating cardiovascular [11], skeletal [12] and performance adaptations [8] as well as changes in psychological quality of life in the study participants [13]. As such, studies from our research group have recently compared the performance effect and cardiac adaptations of recreational soccer with that of resistance training in elderly subjects [8;11]. However, the effects of soccer training in comparison to resistance training on variables such as leg muscle mass, fat mass distribution, muscle anti-oxidative capacity and glucose control have not been investigated in elderly untrained male subjects.

Thus, the aim of the present study was to investigate the long-term effects of soccer training on body composition, anti-oxidative capacity and glucose tolerance and furthermore, evaluate the response on these variables to a similar period of resistance training.

## Materials and Methods

### Subjects

**Fig 1:**
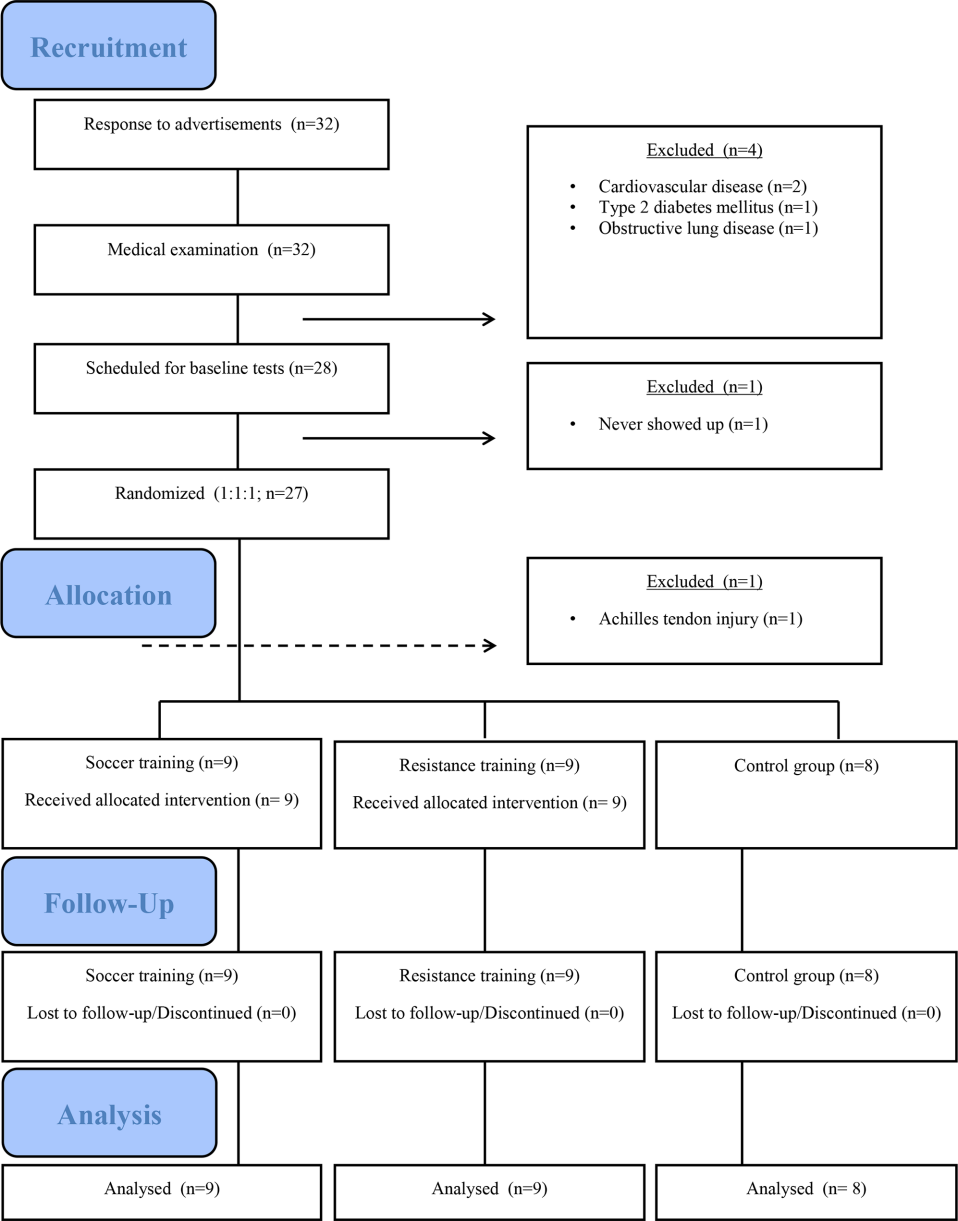
Recruitment flow chart. Flow chart displaying the recruitment process of untrained healthy 65- to 75-year-old male participants. Number of participants after advertisement in local newspapers in the Copenhagen area of Denmark (n = 32), after medical examination (n = 28), randomization (n = 27), group allocation to soccer training (SG; n = 9), resistance training (RG; n = 9), or a control group (CG; n = 8).

Elderly male subjects were recruited via advertisements in local newspapers. Twenty-seven healthy elderly male subjects (age: 68.1±2.1 (range: 63–74) yrs) (mean±SD) were randomly (1:1:1) assigned to either a soccer training group (SG) (n = 10), a resistance training group (RG) (n = 9), or an inactive control group (CG) (n = 8) stratified for body mass index (BMI) and maximal oxygen uptake (VO_2_max) (Fig 1). Medical screening was performed before the start of the intervention period. No subjects took any medication during the study, and none of the subjects were smokers. With the exception of one subject, who was a recreational golfer, none of the subjects had been involved in regular physical exercise training during a major part of their adult life. The participants reported that they had been primarily inactive the past 5–10 yrs. Exclusion criteria were symptoms or history of cardiovascular disease, hyperglycaemia, or diagnosed hypertension. During the initial phase of the intervention period, one subject from the soccer training group left the study due to an Achilles tendon tear, and the data from this subject have been excluded. No group differences were detected in mean pre-intervention values for SG, RG and CG with regard to age (68.0±4.0 (±SD) vs 69.1±3.1 vs 67.4±2.7 yrs), body weight (77.7±9.4 vs 85.8±12.0 vs 89.3±12.4 kg), height (173.3±7.8 vs 176.7±9.8 vs 179.0±6.2 cm), BMI (26.1±3.9 vs 27.4±2.8 vs 27.9±4.6 kg m^-2^) and VO_2_max (27.5±5.4 vs 28.9±5.5 vs 30.8±3.3 mL min^-1^ kg^-1^).

All subjects were informed of potential risks and discomforts associated with the experimental procedures before giving their written informed consent to participate. The study conformed to the code of ethics of the World Medical Association (Declaration of Helsinki) and was approved by the ethical committee for the greater Copenhagen area (De Videnskabsetiske Komiteer for Region Hovedstaden**/**H-1-2011-013). The study was reported at ClinicalTrials.gov.: NCT01530035.

### Experimental design

The subjects in SG and RG were instructed to perform a 1-h training session twice per week for 16 weeks, and three times a week for the following 36 weeks. The subjects in CG continued with their daily routines and did not change lifestyle during the intervention period. In both SG and RG, the training was supervised during the entire intervention period. The soccer training sessions were conducted outdoors from April to November on a 30-45-m-wide and 45-60-m-long natural grass area and consisted of small-sided games (three-a-side, four-a-side or five-a-side). In the event of an uneven numbers of participants, one of the investigators joined in. For the first 12 wks, each training session was initiated with a 15-min low-intensity warm-up, including stretching, and the training was organised as 3x15-min exercise periods with 2-min rest periods. From 13–52 wks, the players perform an individual 5-min warm-up prior to the soccer training followed by 4x15-min exercise periods with 2-min rest periods. Due to seasonal weather variations, the soccer training was performed in an indoor gym with a 20 x 40 m wooden floor from November to March.

In RG, 5 min of low intensity warm-up was followed by resistance training including five exercises: Leg press, seated leg extension, hamstring curl, lateral pull-down and lateral dumbbell raises. Each set of exercises was separated by 1.5 min rest, and at the end of each training session 5 min of core training (crunches, hip extension, side bends, diagonal lifts, and trunk rotation) was performed. The individual training loads were set using the “RM” (repetition maximum) notion for resistance training. The training intensity in each exercise was progressively increased as follows: 3 sets of 16–20 RM (week 0–4), 3 sets of 12 RM (week 5–8), 3 sets of 10 RM (week 9–12) and 4 sets of 8 RM (week 13–52), and the participants were encouraged to perform the exercises with maximal speed during the concentric phase of the movement. From week 25 and onwards, lunges (performed as standing lunges with dumbbells) and seated row (performed as cable pulls) (4 sets of 8 RM) were added to the training prescription. Determination of 16, 12, 10 and 8 RM was carried out in four week intervals.

During the 52-wk intervention, the attendance rate for SG and RG was 66±4% (range: 61–114 training sessions) and 73±3% (77–116 training sessions), respectively. The average number of training sessions per week over the full 52-wk intervention was 1.7±0.3 (1.2–2.2) and 1.8±0.3 (1.4–2.2) in SG and RG, respectively. There was no difference between the training groups (p>0.05). All training and testing sessions were conducted at the University of Copenhagen.

### Measurements and test procedures

#### Habitual dietary intake

Habitual dietary intake was recorded after 0, 16 and 52 wks of the intervention period. The subjects filled out a standardized questionnaire to monitor food intake over a three day period including a day during the week-end. Data were subsequently analysed using a web-based soft-ware (www.madlog.dk). Basal metabolic rate (BMR, MJ/day) was calculated according to the Harris-Benedict equation [14].

#### Habitual physical activity level

At baseline, habitual physical activity level and quality of movement were monitored using pedometer measurements (Yamaxx Digi-Walker, model SW 701; Yamasa Tokei Keiki Co., Ltd., Tokyo, Japan) and a modified Danish version of the Physical Activity Scale-2 questionnaire [15], respectively. Before the start of the intervention period, daily step-count was 6275±1054, 7060±1940, 5986±594 steps/day in SG, RG and CG, respectively, which was not different between the three groups (p>0.05). The daily time spend with moderate (stair climbing, fast walking or similar) was 22±9 vs 28±11 vs 24±6 min/day and the time spend with strenuous physical activity (jogging, aerobics or similar) was 0±0, 0±0, and 0±0 min/day in SG, RG, and CG, respectively, with no differences observed between the three groups (p>0.05). No changes in habitual physical activity levels were reported by any subject during the intervention period besides participation in soccer or resistance training for the subjects in SG (n = 9) and RG (n = 9), respectively.

#### Body composition

Whole body and regional fat mass and lean mass were determined by whole body Dual energy X-ray absorptiometry (DXA) scanning (Prodigy Advance, Lunar Corporation, Madison, Wisconsin, USA). Scanning was performed between 7 and 10 a.m. under standardized conditions after an overnight fast. All DXA scans were performed by the same experienced observer and the DXA software regional cut-points were visually inspected and manually adjusted if necessary. Body height and body weight were measured on a standard scale with subjects wearing light clothes and BMI (kg/m^2^) was subsequently calculated.

#### Oral glucose tolerance testing (OGTT)

An OGTT was performed after 0, 16 and 52 wks of the intervention period under standardized conditions. A glucose bolus of 75 g was ingested in 5 min according to WHO guidelines. Blood samples were collected from an antecubital vein immediately before and again after 15, 30, 60 and 120 min, and subsequently analyzed for glucose concentration. Calculated area under the curve (AUC) for glucose as well as resting and 2 h glucose values are presented.

#### Muscle biopsy and blood sample collection

All invasive procedures were performed 48–72 h after a training session, between 7 and 10 a.m., and under standardized conditions after an overnight fast. A blood sample was collected from an antecubital vein, and a biopsy was collected at rest after 0, 16, and 52 wks from m. vastus lateralis under sterile conditions and local anesthesia (1% Lidocaine, Amgros 742122, Copenhagen, Denmark) using the Bergstrom technique [16]. A part of the muscle sample (40 mg wet weight) was immediately frozen in liquid N_2_ and stored at –80°C. The remainder of the muscle tissue was mounted in an embedding medium (OCT Tissue-Tek, Sakura Finetek, Zoeterwoude, NL) and frozen in pre-cooled isopentane and subsequently stored at –80°C until further analysis.

#### Blood analyses

Whole blood samples were analyzed for basal levels of glycosylated hemoglobin (HbA1c) using liquid chromatography (TOSOH G7). Basal plasma levels of glucose, insulin, cholesterol and triglycerides were analyzed using automated procedures and standard reagents (Cobas Fara, Roche, Neuilly sur Seine, France) at the clinical laboratory at Rigshospitalet, Copenhagen, Denmark. Homeostatic model assessment of insulin resistance (HOMA-IR) was calculated using the Oxford calculator (www.dtu.ox.ac.uk/homacalculator/).

#### Muscle analysis–Maximal enzyme activity

The frozen samples were weighed before and after freeze-drying to determine water content. The samples were then dissected free of all visual connective tissue and blood by light microscopy (Stemi 2000-C, Zeiss, Oberkochen, Germany) at a room temperature of -18°C and a relative humidity below 30%. The muscle tissue of dry weight samples (2 mg) was homogenized (1:400) in a 0.3 M phosphate BSA buffer adjusted to pH 7.7 and phosphofructokinase (PFK), hydroxyacyl-CoA dehydrogenase (HAD), and citrate synthase (CS) muscle enzyme activity was determined fluorometrically as previously described [17].

#### Muscle analysis–Immunofluorescence microscopy

**Fig 2:**
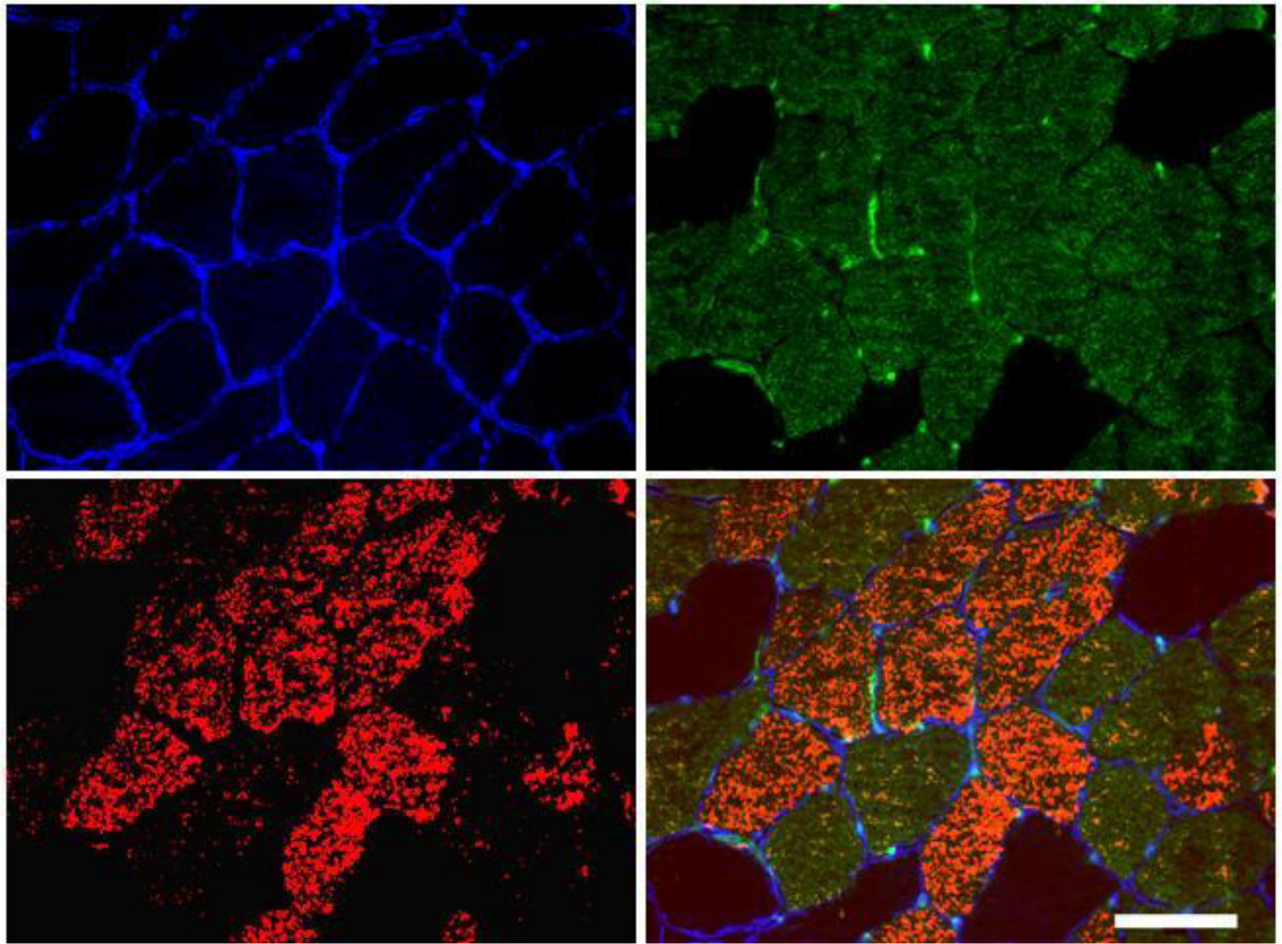
Representative immunofluorescent histochemical staining. Targets shown are; myofibre borders (top left/blue), capillaries (top right/light green) and myofibre type I/IIA (top right/dark green), myofibre type slow/type I (bottom left/red) and a merged over-lay af the above (bottom right). Scale bar = 100 μm.

For determination of muscle fibre cross sectional area, fibre type distribution, and capillarization, the embedded muscle samples were cut using a cryostat, and transverse sections 8 μm in thickness were placed onto glass slides. To verify the cross-sectional orientation of the individual muscle fibre, multiple samples were cut and examined under light microscopy until at cross-section of desirable size, orientation, and uniform polygonal appearance was visible. Only areas without artefacts or tendency to longitudinal cuts were analyzed. Staining targets were visualized pair wise using a standard protocol as previously described [18]. Firstly, capillaries and myofibre type I/IIA were visualized, followed by visualization of myofibre borders and myofibre type slow/type I (Table 1). Specificity of the staining was assessed by single staining, and by staining without the primary antibody. Three individual muscle fibres types were identified as types I (red), IIA (green), and IIX (unstained/black) [19] (Fig 2). Visualization was performed on a computer screen using a light microscope (Carl Zeiss Axio Imager M1, Zeiss, Oberkochen, Germany), and all morphometric analyses were performed using a digital analysis program (Carl Zeiss, AxioVision 4.6). Two or more separate sections of a cross-section were used for analyses, and the cross-sectional area was assessed by manually drawing the perimeter around each selected section. The number of muscle fibres and capillaries within each section was counted, and capillary supply was subsequently expressed as capillaries per fibre (C:F-ratio) and capillary density (capillaries mm^-1^). A mean of 143 myofibres (range: 89–231) were analyzed per biopsy, and mean fibre area was assessed by manual drawing of the perimeter of each muscle fiber. All analyses were carried out manually by the same blinded investigator.

**Table 1:** List of anti-bodies.

**Immunoflourenscence microscopy**		**ID**	**Concentration**	**Manufacturer**
Primary	Capillaries	VECTB-1065	1:100	VWR, Bie & Berntsen, Herlev, Denmark
	Myofibre type I/IIA	BF-35	1:50	Hybridoma Bank, Iowa City, IA, USA
	Myofibre Type I	M8421	1:1000	Sigma-Aldrich, Brondby, Denmark
	Myofibre border/Laminin	Z0097	1:500	Dako, Glostrup, Denmark
Secondary	Streptavidin/FITC	F0422	1:200	Dako, Glostrup, Denmark
	Alexa-488 donkey anti-mouse	A21202	1:1000	Invitrogen, Life Technologies Denmark, Naerum, Denmark
	Alexa-555 donkey anti-mouse	A31570	1:1000	Invitrogen, Life Technologies Denmark, Naerum, Denmark
	Alexa-350 goat anti-rabbit	P10994	1:1000	Invitrogen, Life Technologies Denmark, Naerum, Denmark
**Western blotting**		**ID**	**kDa**	**Manufacturer**
Primary	Superoxide dismutase-2	AB10346	25	Calbiochem, Merck KGaA, Darmstadt, Germany
	Glucose Transporter-4	PA1-1065	42	Thermo Scientific, Hvidovre, Denmark
	Akt-2	3063	60	Cell Signalling Technology, Danvers, MA, US
	Mammalian target of rapamycin (mTOR)	2972	289	Cell Signalling Technology, Danvers, MA, US
	Myostatin/GDF-8	sc-6885-R	26	Santa Cruz Biotechnology, Dallas, TX, US
	Follistatin/H-114	sc-30194	35–70	Santa Cruz Biotechnology, Dallas, TX, US

#### Muscle analysis–Protein expression in muscle homogenate lysates

Changes in protein expression were determined by a standard western blotting procedure as previously described in detail by our laboratory [20]. In short, the freeze dried and dissected muscle tissue was homogenized before the total protein concentration in each sample was determined by a BSA standard kit (Pierce Biotechnology Inc., Rockford, IL, US.). Samples were mixed with 6 x Laemmli buffer (7 ml 0.5M Tris-base, 3 ml glycerol, 0.93 g DTT, 1 g SDS and 1.2 mg bromophenol blue) and ddH_2_0 to obtain the same total protein concentration. From each muscle biopsy two samples were made, and samples from the same subject were loaded on the same gel and the standard western blot procedure was performed. The intensity of each band were first normalized to the mean intensity of two human standard lysates loaded on the same gel and then normalized to the PRE group mean value separately for SG, RG and CG. As previously described [21] outliers were identified and excluded. The exclusion criteria were set to ratio changes from 0 to 16 wks and from 0 to 52 weeks ± 2 SD away from the group mean. As ratios are, by definition, not normal distributed and were log transformed before the statistical analyses and then back transformed to geometric means with the 95% confidence interval. Changes in percent were obtained as the differences in geometric means. The specific antibodies used to determine muscle protein expression are listed in Table 1. The HRP conjugated goat anti-rabbit (4010–05, SouthernBiotech, Birmingham, AL, US) was used as secondary antibody throughout.

### Statistics

Group differences before the intervention period as well as between-group and within-group changes after 16 and 52 wks were analyzed using two-way repeated measures ANOVA. Shapiro-Wilk (normality of the distribution) and Levene’s (homogeneity of variance) tests were calculated before statistical evaluation for all experimental data to justify the application of analysis of variance (ANOVA) statistics. To meet the assumptions for applying general linear models, logarithmical transformation was applied in the case of a significant normality test. The variables OGTT AUC and TC/HDL-cholesterol ratio were separately analyzed with the application of Friedman’s repeated measures ANOVA on ranks. Changes in protein expression were determined using one-way repeated measures ANOVA separately for SG, RG and CG, respectively, based on data being expressed in arbitrary units making comparisons between groups difficult to interpret. When a significant time-by-group effect could be detected a Student–Newman–Keuls (SNK) stepwise multiple comparisons procedure was applied to determine differences in group means between different time points. Also, Tukey’s post-hoc test analysis was applied for comparison. Significant and non-significant (NS) Tukey’s stepwise multiple comparisons procedure p-values are reported. For missing values the last observation carried forward method was used. All analyses were controlled with two-way repeated measures ANOVA without imputations and no relevant statistical differences were found. Data are reported as mean ± SEM. p<0.05 was chosen as the level of significance. Statistical analyses were performed using Sigma plot (Systat Software Inc., San Jose, CA, US) version 11.0.

## Results

### Habitual dietary intake

At baseline, estimated daily energy intake was 9.0±0.4, 8.9±0.2 and 8.6±0.2 MJ/day in SG, RG, and CG, respectively. A positive energy balance was observed in all subjects with a calculated activity factor (daily energy intake/BMR) being 1.4±0.1, 1.3±0.2, and 1.2±0.1 in SG, RG, and CG, respectively. Macro-nutrient distribution of carbohydrate, fat and protein accounted for 47±2, 31±1, 22±1% of total daily energy intake, respectively, in SG, 52±2, 28±2, 20±1%, respectively, in RG, and 50±2, 31±3, 19±1%, respectively, in CG. Daily protein intake was 1.7±0.1, 1.5±0.1, and 1.4±0.1 g/kg/day in SG, RG, and CG, respectively. No group or time effects (SNK; p>0.05 –Tukey; p>0.05) could be detected for estimated daily energy intake, macro-nutrient distribution, calculated activity factor and daily protein intake throughout the intervention period.

### Body composition

BMI at baseline was not different in SG, RG, and CG (Table 2). In SG, BMI was reduced by 1.5±0.5% (SNK; p<0.05 –Tukey; NS) and 3.0±0.9% (SNK; p<0.001 –Tukey; p<0.001) after 16 and 52 wks, respectively, whereas it was not changed in RG (Table 2). In CG, BMI was unchanged after 16 wks and 1.8±1.4% higher (SNK; p<0.05 –Tukey; p<0.05) after 52 wks compared to after 16 wks, respectively.

**Table 2:** Anthropometric characteristics during 52 wks of soccer or resistance training in elderly men.

	SG			RG			CG		
	0 weeks	16 weeks	52 weeks	0 weeks	16 weeks	52 weeks	0 weeks	16 weeks	52 weeks
Total body mass (BM) (kg)	77.6±3.1	76.5±3.1	75.3±3.1***[***]	85.8±4.0	85.4±3.9	85.5±4.0	89.3±4.4	88.7±4.2	90.3±4.1§#[§#]
BMI (kg/m^2^)	26.0±1.2	25.6±1.2*	25.2±1.1***§[***]	27.4±0.9	27.3±1.0	27.3±1.0	28.0±1.6	27.8±1.5	28.3±1.6§ [§]
Lean body mass (LBM) (kg)	53.0±1.1	53.0±1.2	52.4±1.2	56.4±1.7	57.6±1.7*# [*]	57.3±1.8#	59.1±1.8#[#]	59.1±1.6#[#]	59.5±1.7#[#]
Upper body lean mass (kg)	31.7±0.6	31.2±0.7	31.1±0.4	32.5±0.9	33.4±1.0*[*]	33.1±1.0	35.3±1.3	35.5±1.3#[#]	35.9±1.5#[#]
Leg lean mass (kg)	17.7±0.5	18.1±0.6	17.5±0.6§ [§]	20.0±0.9	20.3±0.8	20.2±0.9#[#]	19.9±0.7	19.5±0.5	20.1±0.6§#[§#]
Total fat mass (kg)	21.6±2.5	20.5±2.6	19.8±2.5	25.8±2.6	24.4±2.5	24.8±2.6	26.7±2.9	26.1±2.9	26.6±2.7
Leg fat mass (kg)	6.6±0.7	6.2±0.8	6.0±0.7	7.3±0.6	6.8±0.6	6.8±0.6	7.1±0.8	6.8±0.8	6.9±0.8
Upper body fat mass (kg)	14.3±1.8	13.6±1.9	13.1±1.7	17.9±2.1	16.9±2.0	17.1±2.0	18.8±2.2	18.5±2.2	20.6±2.7
Body fat (%)	27.2±2.3	26.1±2.5	25.6±2.4	29.4±2.3	27.9±2.1	28.2±2.3	29.4±1.8	29.2±1.8	29.8±1.6
Android fat (%)	37.1±3.0	35.1±3.1	34.7±2.6	40.2±3.6	38.7±3.1	38.1±3.4	41.4±2.1	40.8±2.1	40.8±1.9
Gynoid fat (%)	31.0±2.1	29.2±2.5	30.3±2.4	31.5±2.0	29.6±1.5	30.0±1.8	29.8±1.4	29.6±1.5	28.5±1.3
A/G-ratio	1.20±0.07	1.22±0.07	1.15±0.05	1.27±0.11	1.31±0.09	1.27±0.10	1.40±0.07	1.39±0.06	1.45±0.07#[#]

Anthropometric characteristics in elderly male subjects before (0 wks) as well as after 16 and 52 wks of soccer training (SG), resistance training (RG) or continuation of an inactive lifestyle (CG).Means ± SEM are presented. SNK:

* Significantly (p<0.05) different from 0 wks.

*** Significantly (p<0.01) different from 0 wks.

§ Significantly (p<0.05) different from 16 wks.

# Significantly different from the corresponding SG value (p<0.05). Tukey: [Brackets around notation].

Total lean body mass was lower (SNK; p<0.05 –Tukey; p<0.05) at baseline in SG compared to CG (Table 2). In SG, leg lean mass was lower (SNK; p<0.05 –Tukey; p<0.05) after 52 wks than after 16 wks, and not different compared to 0 wks, whereas total and upper body lean mass was not different after 52 wks compared to 0 and 16 wks (Table 2). In RG, upper body lean mass was higher (SNK; p<0.05 –Tukey; p<0.05) after 16 wks compared to 0 wks (Table 2), whereas leg lean mass was unchanged throughout the intervention period (Table 2). In CG, total, upper body and leg lean mass remained the same during the intervention period, whereas leg lean mass was higher (SNK; p<0.05 –Tukey; p<0.05) after 52 wks than after 16 wks, and not different compared to after 0 wks.

Total, leg and upper body fat mass as well as android, gynoid, and whole body fat percentage was not different in SG, RG, and CG at baseline, and did not change during the intervention period (Table 2). A/G-ratio after 52 wks was lower (SNK; p<0.05 –Tukey; p<0.05) in SG compared to CG (Table 2).

### Muscle variables

Maximal activity of CS, HAD and PFK at baseline was not different in SG, RG and CG, and did not change during the intervention period (Table 3). Likewise, muscle fibre type distribution, fibre area and capillary density at baseline were not different between SG, RG and CG, and no changes were observed during the intervention period (Table 3).

**Table 3:** Muscle variables during 52 wks of soccer or resistance training in elderly men.

	SG			RG			CG		
	0 weeks	16 weeks	52 weeks	0 weeks	16 weeks	52 weeks	0 weeks	16 weeks	52 weeks
CS (μmol/g dw/min)	19.5±1.5	24.8±2.3	26.9±1.6	25.4±1.4	28.9±1.5	26.1±1.9	22.0±1.4	23.9±2.3	23.8±2.5
HAD (μmol/g dw/min)	16.9±1.0	18.0±1.3	18.7±0.9	19.2±0.8	19.5±1.0	18.1±1.3	16.8±0.7	17.9±1.5	18.8±1.7
PFK (μmol/g dw/min)	251±14	276±19	258±9	235±25	271±15	252±21	258±18	250±27	241±14
Capillary density (cap/mm^2^)	343±29	342±28	329±19	312±15	306±15	256±7	300±20	290±25	293±21
Capillary density (C:F-ratio)	1.36±0.07	1.39±0.15	1.35±0.07	1.51±0.12	1.62±0.13	1.54±0.11	1.57±0.06	1.46±0.12	1.63±0.17
Type I area (μm^2^)	4258±497	4082±326	4317±292	5066±423	5516±530	6157±658	5478±389	5342±637	5917±539
Type IIA area (μm^2^)	3966±480	4163±534	3877±272	4877±423	5043±455	5770±527	5355±537	5290±703	5406±605
Type IIX (μm^2^)	3933±691	3550±405	3427±410	4277±544	5103±720	5618±402	4664±606	4683±745	4522±513
Mean fibre area (μm^2^)	4148±465	4096±361	4161±232	4910±410	5384±537	6093±585	5438±493	5295±652	5579±506
% Type I (no. of fibres)	46±5	43±3	51±3	49±4	49±5	52±4	43±4	41±3	42±4
% Type IIA (no. of fibres)	38±3	42±4	38±4	38±2	40±4	40±4	40±4	48±4	42±6
% Type IIX (no. of fibres)	16±3	14±2	10±2	14±3	12±2	8±3	16±5	12±2	16±4

Muscle enzyme activity, capillary density, fibre area, and fibre type distribution in elderly male subjects before (0 wks) as well as after 16 and 52 wks of soccer training (SG), resistance training (RG) or continuation of an inactive lifestyle (CG). Means ± SEM are presented.

**Fig 3:**
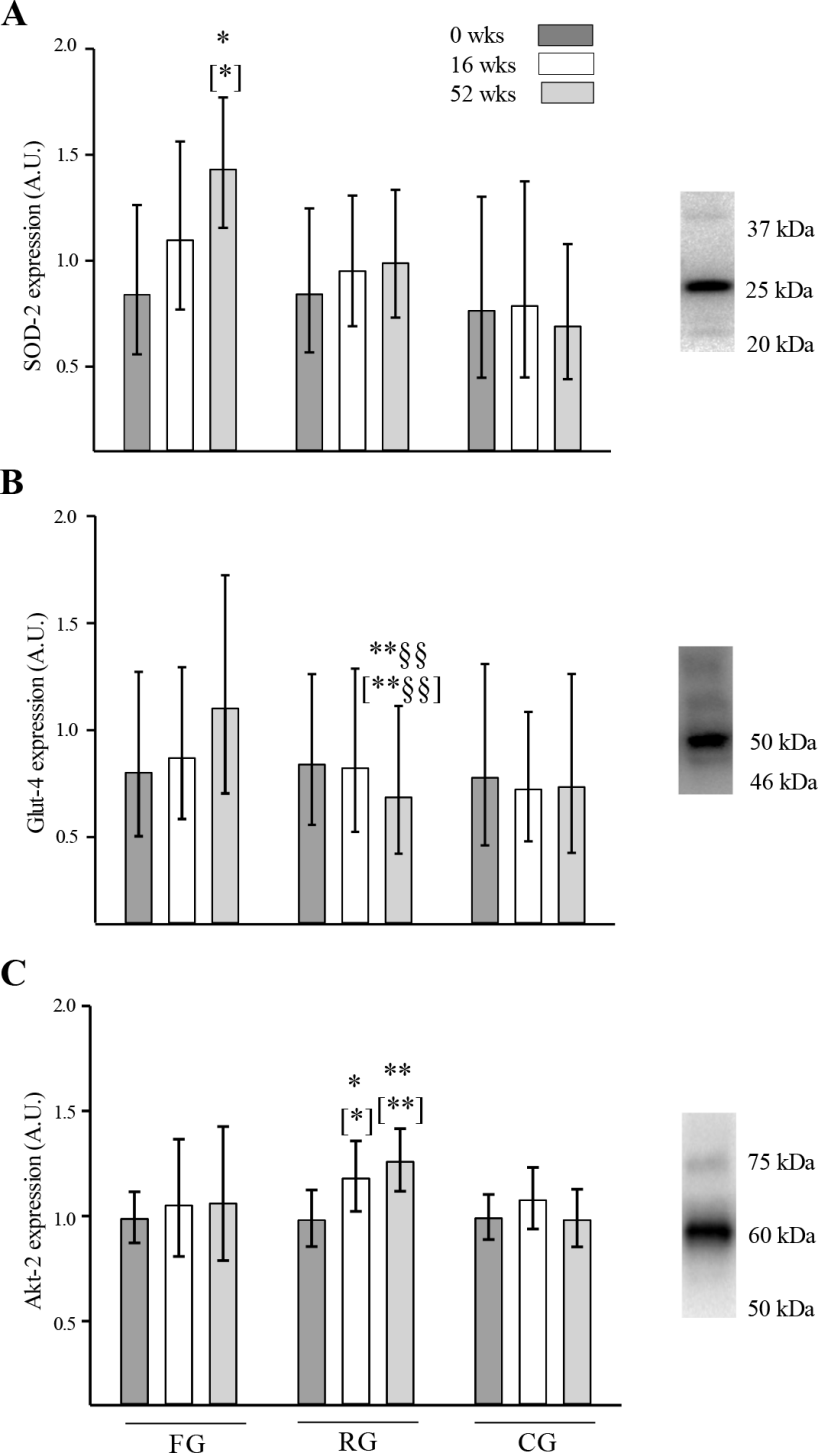
Protein expression in muscle homogenate lysates. Expression (A.U.: arbitrary units) of (A) superoxide dismutase-2 (SOD-2), (B) glucose transporter-4 (Glut-4), and (C) Akt-2 in elderly subjects after 0 (dark bars), 16 (white bars) or 52 wks (grey bars) of soccer training (SG), resistance training (RG) and continuation of an inactive lifestyle (CG). Geometric means and 95% confidence interval are presented. SNK: * Significantly (p<0.05) different from 0 wks. ** Significantly (p<0.01) different from 0 wks. §§ Significantly (p<0.01) different from 16 wks. Tukey: [Brackets around notation].

**Fig 4:**
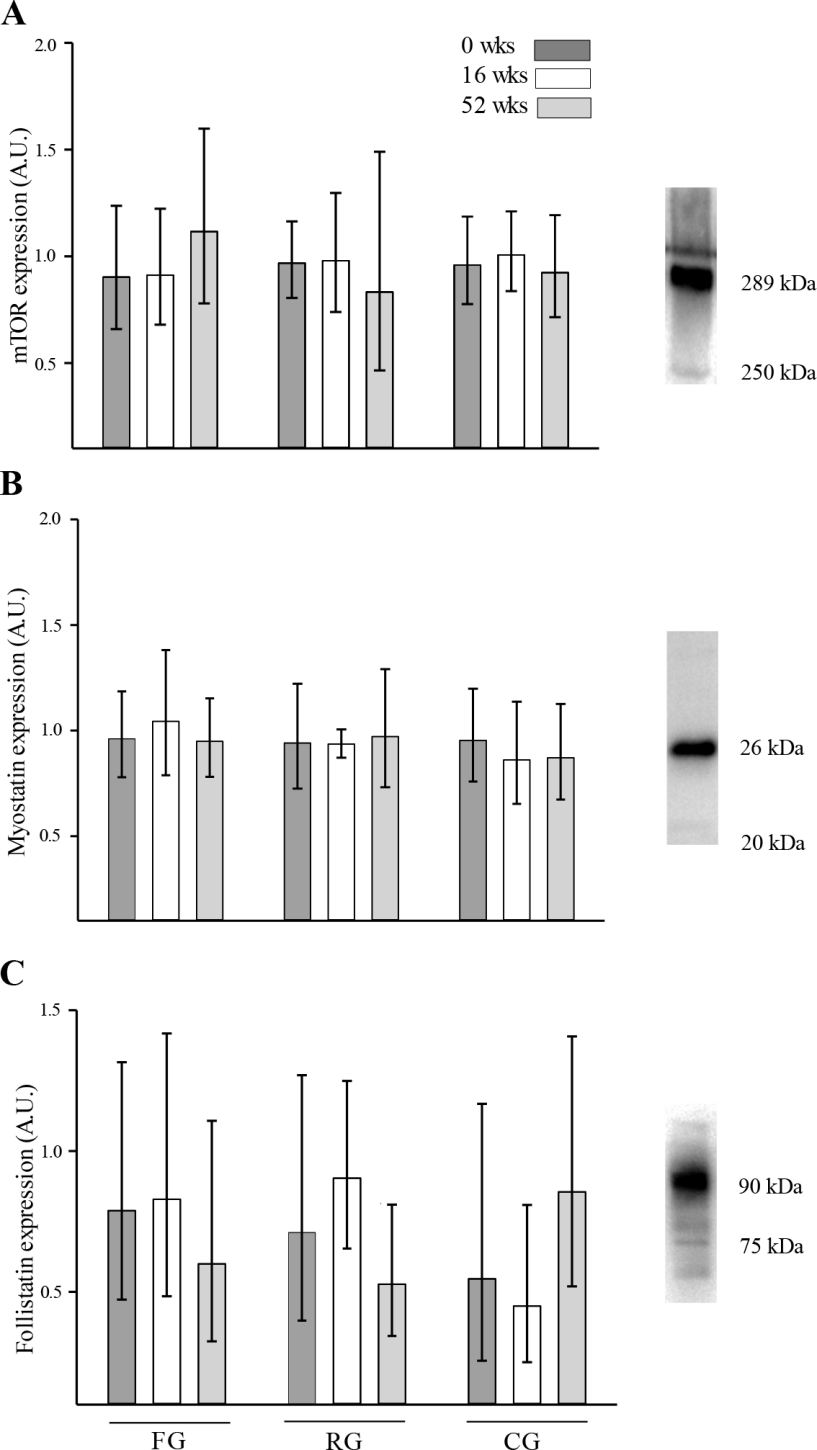
Protein expression in muscle homogenate lysates. Expression (A.U.: arbitrary units) of (A) mammalian target of rapamycin (mTOR), (B) myostatin, and (C) in elderly subjects after 0 (dark bars), 16 (white bars) or 52 wks (grey bars) of soccer training (SG), resistance training (RG) and continuation of an inactive lifestyle (CG). Geometric means and 95% confidence interval are presented.

In SG, the expression of SOD-2 was higher (59%; SNK; p<0.05 –Tukey; p<0.05) after 52 wks compared to 0 wks, whereas in RG and CG no change was observed during the intervention period (Fig 3A). In SG, Glut-4 expression tended to be higher (30%; SNK; p = 0.076 –Tukey; NS) after 52 wks compared to 0 wks, whereas Glut-4 was lower (15%; SNK; p<0.01 –Tukey; p<0.01) in RG after 52 wks compared to 0 wks and 16 wks (Fig 3B). Akt-2 expression did not change during the intervention period in SG, whereas in RG, it was 20% higher (SNK: p<0.05 –Tukey; p<0.05) after 16 wks and 28% higher (SNK; p<0.01 –Tukey; p<0.01) after 52 wks compared to 0 wks (Fig 3C). In SG, the expression of mTOR tended to be higher (21%, p = 0.085) after 52 wks compared to 0 wks, whereas it did not change in RG (Fig 4A). The expression of myostatin did not change during the intervention period in neither of the groups (Fig 4B). The expression of follistatin did not change in SG during the intervention period, whereas it tended to be lower (38%; SNK; p<0.054 –Tukey; NS) in RG after 52 wks compared to after 16 wks (Fig 4C). In CG, the expression of SOD-2, Glut-4, and Akt-2 (Fig 3A–3C), as well as the expression of mTOR, myostatin, and follistatin (Fig 4A–4C), did not change during the intervention period.

### Fasting blood glucose, plasma insulin, OGTT, and HbA1c

Fasting blood glucose, HbA1c, plasma insulin, calculated HOMA-IR and 2 h OGTT blood glucose levels at baseline were not different in SG, RG, and CG, and they did not change in either group throughout the intervention period. In SG, the area under the curve (AUC) for glucose was 16.3% (SNK; p<0.05 –Tukey; p<0.05) after 16 wks compared to 0 wks, and higher (SNK; p<0.05 –Tukey; NS) after 52 wks compared to after 16 wks (Table 4).

**Table 4:** Resting blood variables during 52 wks of soccer or resistance training in elderly men.

	SG			RG			CG		
	0 weeks	16 weeks	52 weeks	0 weeks	16 weeks	52 weeks	0 weeks	16 weeks	52 weeks
Fasting blood glucose (mM)	5.1±0.1	5.2±0.2	5.1±0.2	5.2±0.1	5.1±0.2	5.3±0.2	5.5±0.2	5.6±0.2	5.7±0.1
2 h OGTT blood glucose (mM)	7.2±1.4	6.7±1.2	7.1±1.5	5.5±1.1	6.4±1.6	5.9±0.9	6.9±1.1	7.1±1.2	6.9±1.5
OGTT AUC (mM/2 hrs)	16.5±1.6	14.2±1.4*[*]	16.0±1.3§	14.8±0.6	15.1±0.9	15.3±0.5	15.8±1.7	16.2±1.0	16.7±0.8
HbA1c (mM)	5.3±0.1	5.4±0.1	5.3±0.1	5.5±0.1	5.5±0.1	5.5±0.1	5.7±0.1	5.8±0.1	5.7±0.1
Fasting insulin (pM)	33±5	26±3	24±2	41±6	35±4	36±5	48±10	40±7	48±13
HOMA-IR	1.3±0.2	1.0±0.1	0.9±0.1	1.6±0.3	1.3±0.2	1.4±0.2	2.0±0.5	1.7±0.3	2.0±0.6
Total cholesterol (mM)	5.6±0.4	5.5±0.3	5.7±0.3	5.2±0.3	4.9±0.2	5.3±0.4	5.5±0.3	5.2±0.2	5.6±0.3
HDL cholesterol (mM)	1.6±0.1	1.7±0.1	1.8±0.1	1.4±0.1	1.5±0.1	1.7±0.2	1.6±0.1	1.6±0.1	1.6±0.1
LDL cholesterol (mM)	3.5±0.3	3.3±0.2	3.5±0.3	3.1±0.2	3.0±0.2	3.3±0.2	3.3±0.2	3.1±0.2	3.4±0.3
Triglycerides (mM)	0.9±0.1	1.0±0.1	0.9±0.1	1.1±0.1	1.2±0.1	1.2±0.2	1.0±0.1	1.1±0.2	1.2±0.3
Total cholesterol/HDL cholesterol	3.7±0.3	3.4±0.2*	3.2±0.3*[*]	3.7±0.2	3.5±0.2*	3.2±0.2*§[*]	3.6±0.3	3.3±0.3	3.5±0.3
LDL cholesterol/HDL cholesterol	2.3±0.2	2.0±0.2	2.0±0.2	2.2±0.2	2.1±0.2	2.0±0.2	2.2±0.2	2.0±0.2	2.1±0.3

Resting blood glucose, 2h Oral Glucose Tolerance Test (OGTT), blood glucose, HbA1c, plasma insulin and total cholesterol (TC), cholesterol sub-fractions as well as calculated homeostatic model assessment of insulin resistance (HOMA-IR) in elderly male subjects before (0 wks) as well as after 16 and 52 wks of soccer training (SG), resistance training (RG) or continuation of an inactive lifestyle (CG). Means ± SEM are presented. SNK:

* Significantly (p<0.05) different from 0 wks.

§ Significantly (p<0.05) different from 16 wks.

Tukey: [Brackets around notation].

### Blood lipid variables

Plasma triglycerides, total HDL and LDL cholesterol were not different at baseline in SG, RG, and CG, and did not change during the intervention period in either group. In SG, the ratio between total and HDL cholesterol (TC:HDL-ratio) was 8±2% (SNK; p<0.05 –Tukey; NS) and 12±3% (SNK; p<0.05 –Tukey; p<0.05) lower after 16 and 52 wks compared to 0 wks, respectively. In RG, the TC:HDL-ratio was lower after 16 and 52 wks (5±2% (SNK; p<0.05 –Tukey; NS) and 14±3% (SNK; p<0.05 –Tukey; p<0.05), respectively) compared to 0 wks, and also lower (SNK; p<0.05 –Tukey; NS) after 52 wks compared to after 16 wks. In CG, the TC:HDL-ratio did not change throughout the intervention period (Table 4).

## Discussion

The major findings of the present study were that 52 wks of regular soccer training performed as small-sided games in elderly men lead to decreases in BMI and increases in SOD-2 expression along with a reduced ratio between total and HDL cholesterol as well as an improved response to a standardized glucose challenge after 16 weeks of the intervention period. In addition, a 52-wk period of resistance training resulted in higher total and upper body lean mass with no changes in anti-oxidative potential and glucose tolerance. Furthermore, several factors, such as muscle oxidative enzyme activities and fibre type area, did not change in neither the soccer nor the resistance training group during the intervention period, although the training significantly impacted the cardiovascular and muscular systems [8].

In the soccer training group SOD-2 expression was 59% higher after 52 wks of training. In accordance, it has been observed that basal SOD-2 expression is higher and oxidative stress is lower in endurance-trained compared to untrained elderly [22]. In the soccer training group, the average heart rate during the soccer training was around 80% of HRmax and for 17% of a training session heart rate was above 90% of HRmax [8]. The high aerobic loading during soccer training may as such have been the driving force for the elevated SOD-2 expression observed in the soccer training group. This is in agreement with a study investigating the effects of 8 wks of high-intensity aerobic training in elderly subjects observing an 30% increase in SOD-2 expression [23]. Thus, endurance and soccer training appears to be effective in improving skeletal muscle SOD-2 expression in elderly men. In contrary, SOD-2 was unchanged (15%; p>0.05) in the resistance training group after 52 wks. A higher training volume or a longer training period of resistance training may be needed to elicit changes in by SOD-2 expression.

It was an unexpected observation that the maximal activity of CS was unaffected by training (Table 3), despite the fact that the CS activity prior to the intervention period was low and at a similar level to that of other untrained subjects [24]. The soccer training was performed with a high aerobic loading [8], and further, the subjects trained for 1 h per session and training frequency increased from 2 to 3 sessions per week after 16 wks of the intervention period. As such, this finding is in contrast to observed increases in CS activity after 12 wks of soccer training in untrained men [25] or resistance training [26]. Generally, a limited muscular response to training was observed in the participating subjects, and also maximal HAD activity and capillary density were the same after 52 wks compared to before the intervention. It might be speculated, that the activity profile during recreational soccer with more than two-thirds of the training time being spent with standing, walking and jogging [27], does not provide a sufficient training stimulus to induce muscular adaptations in old untrained men. Also, 52 wks of resistance training conducted with high loads and relatively few repetitions did not create changes in oxidative capacity. Thus, the muscular training response appears inhibited in elderly subjects [28] and stronger and/or different stimuli may be needed to create significant changes in muscle oxidative enzymes.

The OGTT response in the soccer training group was better after 16 wks compared to before the intervention period (Table 4). This suggests an improved glucose control. Glut-4 is considered to be the most important glucose transporter in skeletal muscle and is thought responsible for both insulin-stimulated and contraction-induced glucose uptake [29]. Thus, the augmented OGTT response may partly be related to an increase in glucose transport capacity as evaluated by the expression of GLUT-4, even though only a tendency (p = 0.07) was observed in the soccer training group after 52 wks. However, there does not appear to be a close link, as the OGTT response was the same, and the Glut-4 expression was lower in the resistance training group after 52 wks compared to before the intervention period. This is in contrast to findings in other studies assessing the effect of resistance training on glucose tolerance in elderly subjects with impaired glucose tolerance observing increases in GLUT-4 expression and glucose tolerance [30]. Fifty-two weeks of soccer training did not lead to changes in fasting glucose, insulin, and HbA1c (Table 4), which may reflect that the subjects at baseline had a HOMA-IR which was close to the ideal level (HOMA-IR = 1) suggesting a normal glucose metabolism (Table 4). Nevertheless, the soccer training group had a non-significant (p>0.05) 27% reduction in fasting insulin after 52 wks compared to before training, which was greater (p<0.05) than the 12% non-significant (p>0.05) reduction observed in the resistance training group. Lowered fasting insulin may reflect a diminished need for insulin to control the blood glucose concentration. In accordance, the calculated odds ratio of having a positive change in glycaemic control after 24 wks of soccer training was 2.3 (95% CI: 0.34 to 16.2) in middle-aged type 2 diabetic subjects compared to a control group [31]. Altogether, soccer training appears to be an effective training method to positively change glucose control and favorably impact the response to a standardized glucose challenge during the initial phase of a training period even in elderly men with no established indications of impaired glucose metabolism.

In the soccer training group, lean body mass after 52 wks was not different from baseline values. Other studies have shown increases in lean body mass ranging from 1.0 to 1.7 kg after 12–16 wks of soccer training in untrained [25]. This was also the case in the initial phase (wks 0–16) of the present investigation, and it is unclear why the lean body mass decreased in the soccer training group during the last 36 wks of soccer training. Also, it is not clear why a decrease in BMI was observed in the soccer training group after 52 wks despite a reported positive energy balance, but it may be that the methods used to monitor energy intake and consumptions are inaccurate. After 16 wks of resistance training, total body lean mass was 2% higher compared to baseline levels, with no further changes observed after 52 wks (Table 2). The increase in lean body mass was due to a rise in upper body lean mass which is likely to reflect the fact that the resistance training involved a number of exercises specifically confined to the upper extremity. The reason for the lack of improvement in lean mass of the lower body training is not known, however, different muscle groups tend to respond differently to training [32]. Mean fibre size of the vastus lateralis was unaltered in both the soccer training and the resistance training group during the study period. In young men, average muscle fibre size has been shown to increase by 15% after 12 wks of soccer training [25], which is of similar magnitude to the change observed in old men after heavy resistance training [33]. One of the reasons for the lack of increase in muscle mass may be related to insufficient intake of proteins. The daily protein intake of the participants was higher than the recommendation of the American Dietetic Association of 0.8 g/kg/day for elderly people, but there is evidence that larger amounts of protein are required during a period with exercise training [34]. The lack of effect on the muscle mass may also have been influenced by the timing of protein intake in relation to the training session, which was not controlled [35]. Collectively, after 52 wks of regular soccer training, total and leg lean mass were maintained in elderly men, whereas resistance training increased upper body lean mass without any change in body mass.

The present study also investigated the expression of Akt-2 and mTOR, which are related to muscle tissue hypertrophy signaling [36]. Akt-2 expression was unchanged in the soccer training group, whereas it was higher in the resistance group after 52 wks. Thus, resistance training may have impacted muscle signaling cascades involved in muscle growth and repair. However, the expression of mTOR, a key hypertrophic regulator and a down-stream target of Akt, was not changed in either group during the intervention period. This might in part explain the absent hypertrophic response in the leg muscles observed in the present study. It should though be underlined that the biopsies were obtained several days after the last training, and it cannot be excluded that Akt-2 and mTOR was transiently elevated after each training session [37]. Follistatin expression did not change in the soccer training group during the intervention period, whereas it was lower in the resistance training group after 52 wks compared to baseline. This was a surprising finding, as follistatin antagonizes the actions of myostatin, which is known as a powerful negative regulator of muscle growth [38]. However, myostatin abundance may not reflect myostatin activity, and the present study did not distinguish between active and inactive, latent states of myostatin. Thus, in the resting state, the expression of hypertrophic regulators was not altered during a period of soccer training, whereas resistance training changed the basal expression of such proteins.

The concentrations of HDL, LDL, total cholesterol (TC) and triglycerides in the blood did not change during the intervention period in either group. The lack of change may be due to the blood cholesterol and triglyceride concentrations being close to an optimal level before the study [39]. It should be noted, that the TC:HDL-ratio in the soccer raining group was 8 and 12% lower after 16 and 52 wks, respectively, compared to before the intervention period, with comparable changes observed in the resistance training group. TC-HDL-ratio has been reported to contribute to 32% of the calculated population-attributable risk [40], and such changes may therefore play an essential role in the prevention of cardiovascular disease. However, the clinical effect of a reduction in TC-HDL-ratio in a group of healthy elderly subjects without cardiovascular disease is unknown.

The SNK and Tukey’s post hoc analysis approaches were both applied following the finding of a significant ANOVA test. The SNK method uses different critical values for different pairs of mean comparisons unlike the Tukey’s test. The SNK procedure is therefore more likely to incorrectly reject a true null hypothesis and to falsely reveal significant differences between group means (known as a statistical type I error). Hence, the former method is considered very powerful but less conservative than the latter [41]. When a more conservative analytical approach was taken, it appears reasonable to state, that longer training periods (+16 wks) is needed to ensure adaptations in some (e.g. blood lipids) but not all of the tested variables of the present study.

In summary, long-term soccer training positively impacted the skeletal muscle anti-oxidative potential in elderly untrained men with little or no prior experience with soccer training. Also, soccer training favorably altered glucose control in the initial part of the training period, and maintained lean body mass throughout the intervention despite a marked reduction in body mass and BMI. Furthermore, long-term resistance training appeared effective in increasing upper body lean mass and changing the expression of major regulatory signaling proteins.

## Supporting Information

S1 CONSORT Checklist CONSORT 2010 Checklist. (PDF)

S1 Protocol Original study protocol in Danish language. (PDF)

S2 Protocol Translated study protocol in English language. (PDF)

S1 Table Minimal data set underlying the findings in the present study including individual values on anthropometric characteristics, as well as on blood and muscle characteristics in elderly subjects after 0, 16 and 52 wks of soccer training (SG), resistance training (RG) and continuation of an inactive lifestyle (CG). (PDF)
