# Supplementary material for: The Effects of 52 Weeks of Soccer or Resistance Training on Body Composition and Muscle Function in +65-Year-Old Healthy Males – A Randomized Controlled Trial
Source: PLoS One. 2016 Feb 17;11(2):e0148236. doi: 10.1371/journal.pone.0148236 (PMC4757560; doi:10.1371/journal.pone.0148236)
Supplement: S1 Protocol — (PDF) [file pone.0148236.s002.pdf]

De Videnskabsetiske Komitéer
for Region Hovedstaden
Regionsgården
Kongens Vænge 2
3400 Hillerød

København, den 1. april 2011

Anmeldelse til de Videnskabsetiske Komitéer

Alle oplysninger på denne blanket kan blive offentliggjort.

Komité

Primærkomité:	De Videnskabsetiske Komitéer for Region Hovedstaden
Sekundærkomitéer:	
Anmeldelsesnr.:	H-1-2011-013

A. Forsøgsansvarlig

1. Titel:	Læge, PhD
2. Navn:	Charlotte Suetta
3. Adresse:	Bakkegårds Allé 16, st th
4. Postnummer/by:	1804 Frederiksberg C
5. Telefonnr.:	23 33 94 93 (privat) / 24 83 40 37 (BBH)
6. E-mail:	csuetta@gmail.com

B. Evt. anden kontaktperson

1. Titel:	Lektor, PhD
2. Navn:	Peter Krustrup
3. Adresse:	Strandgårds Allé 63
4. Postnummer/by:	3600 Frederikssund
5. Telefonnr.:	38711054 (privat)/ 26 15 43 41 (IFI)
6. E-mail:	pkrustrup@ifi.ku.dk

C. Projektinformation

1. Projekttitel:	**Fodbold og styrketræning som motionsformer hos 65-75 årige mænd: indflydelse på muskelmasse, neuromuskulær funktion og kardiovaskulære risikofaktorer.**
2. Projektets hovedformål:	Hovedformålet er at bidrage med ny viden om de træningsmæssige og sundhedsmæssige effekter af fodbold og styrketræning for ældre mænd. Der gennemføres dels et randomiseret, kontrolleret træningsforsøg over 12 måneder med deltagelse af 60 mænd i alderen 65-75 år (20 kontroller, 20 i fodboldgruppe og 20 i styrkegruppe). De muskuloskeletale og kardiovaskulære effekter undersøges ved måling af fiberarealer, myogene satellitceller, maximal muskelstyrke incl RFD og power, postural balance, knogletæthed, såvel som kondital, blodtryk, kolesterol, triglycerid, insulinfølsomhed og fedtprocent. Tillige gennemføres et tværgående studie hvor samme målinger gennemføres på 20 mænd i alderen 65-75 år med livslang fodbolddeltagelse.
3. Sted(er) for gennemførelse:	Kbh. Universitet, Rigshospitalet, Bispebjerg Hospital.
- Adresse(r) / forsøgsansvarlige	Institut for Idræt, Afd. For Human Fysiologi, Københavns

	Universitet. Universitetsparken 13, 2100 København Ø.<br>Forsøgsansvarlig: Lektor, PhD, Peter Krustrup
	Bispebjerg Hospital, Klinisk Fysiologisk og Nuklear Med Afd., Bispebjerg Bakke 23, Opg.60, 2400NV<br>Forsøgsansvarlig: Læge Charlotte Suetta
	Institut for Idræt og Klinisk Biomekanik, Syddansk Universitet, Odense<br>Forsøgsansvarlig: Prof. Per Aagaard
4. Forsøgsgrupper:	Myndige habile
5. Design:	Randomiseret
6. Biobank forsøg:	Nej
7. Lægemiddelforsøg:	Nej
8. Ekstern økonomisk støtte:	Ja
9. Udbetales der vederlag til fp:	Ja
10. Projekt iværksættes den:	01-04-2011
11. Projekt afsluttes den:	01-10-2012
12. Forventet antal fp:	80
13. Heraf raske:	80
14. Læge/sundhedsfagligt omr.:	Fysisk træning/gerontologisk
15. Sygdommens art/navn:	
16. ICD10 kode:	
17. MedDRA kode:	
18. Multistat projekt:	Nej

Underskrift

Læge, PhD, Charlotte Suetta
Bakkegårds Allé 16, st th, 1804 Frederiksberg

København, 1. april 2011

Anmeldelse til Videnskabsetisk Komité
De Videnskabsetiske Komitéer for Region Hovedstaden
Regionsgården, Kongens Vænge 2, 3400 Hillerød

Projektbeskrivelse

Fodbold og styrketræning som motionsformer hos 65-75 årige mænd: indflydelse på muskelmasse, neuromuskulær funktion og kardiovaskulære risikofaktorer.

Formål

Forsøgets formål er at kortlægge og sammenligne de muskulo-skeletale, neuromuskulære og kardio-vaskulære effekter af henholdsvis fodbold og styrketræning hos utrænede ældre (65-75 årige) mænd. I studiet undersøges blandt andet fiberarealer, satellitceller, muskelstyrke, postural balance, knogletæthed, kondital, blodtryk, kolesterol, triglycerid, insulinfølsomhed og fedtprocent hos 60 mænd der deltager i et kontrolleret, randomiseret træningsstudie over 12 måneder og dels hos 20 mænd i alderen 65-75 år som har deltaget i regelmæssig fodboldtræning hele livet.

Baggrund

Det er veldokumenteret at der ved stigende alder sker et tab i muskelmasse med deraf følgende nedgang i motorisk funktion, og at disse tab accelereres fra omkring 65-års alderen (Aagaard et al. 2010). Dette fald i aktiv muskelmasse og tab i selvhjulpen mobilitet fører til en endnu højere grad af inaktivitet. Tilsammen leder disse faktorer til en øget risiko for sygdom i kredsløb, muskulatur og knogler der bl.a. manifesterer sig i en øget incidens af type II og type I diabetes, forhøjet blodtryk, knogletab, og forringet postural balance. Det er selvsagt af stor betydning for enkelte ældre person at forebygge en sådan udvikling. Samtidig er Danmark – i lighed med resten af den vestlige verden – i færd med at ændre aldersdemografi i form af et stærkt stigende antal ældre over 65 år. Dette gør forebyggelsen af aldersrelateret muskeltab og nedgang i neuromotorisk funktion til et samfundsøkonomisk anliggende, der har særlig høj prioritet.

I Danmark rapporterer 20-26% af de ældre i aldersgruppen 60-66 år at de har problemer med at bevæge sig rundt ved egen hjælp, og 48-77% af aldersgruppen +80 rapporterer om tilsvarende problemer (Skelton & Todd 2004) og det vides at nedsat bevægelsesfunktion udgør en høj risikofaktor for fald hos ældre (Skelton & Todd 2004). I 2004 så man således omtrent 41.000

henvendelser på de danske skadestuer, der var relateret til faldulykker hos ældre (+65 år), heraf førte ca. 12.000 til en indlæggelse (NIPH 2005). Forebyggelse af nedsat bevægelsesfunktion vil således ikke alene være af stor personlig værdi for den enkelte ældre, men også have høj samfundsøkonomisk værdi.

Den nedsatte bevægelsesfunktion ved stigende alder skyldes dels degenerative forandringer i nervesystemet (Aagaard et al. 2010) og dels et progressivt tab af skeletmuskulatur ('sarcopeni'), som accelerer i 60-65 års alderen (Lexell et al. 1988, Frontera et al. 2000, Vandervoort 2000). Muskeltabet skyldes dels en reduktion i antallet af muskelfibre samt et reduceret tværsnitsareal (atrofi) af de tilbageværende muskelfibre (Lexel et al. 1983, 1988), hvilket specielt ses for de hurtigt kontraherende 'explosive' type II muskelfibre (Lexell et al. 1988, Andersen 2003). Dertil kommer at den aldrende skeletmuskulatur viser en nedsat kraftudvikling udtrykt pr tværsnitsareal (Macaluso et al. 2002) indikerende en nedsat muskelkvalitet. Sænkningen i specifik kraftudvikling (↓ kraft / cm^2 muskel areal) skyldes dels nedsat maximal MU fyringsfrekvens (Kamen & Knight 2004, Klass et al. 2008, Christie & Kamen 2010) og dels en reduceret specifik kraftudvikling (N/mm^2) i isolerede muskelfibre som følge af en reduceret koncentration af kontraktile myosin proteiner (Canepari et al. 2010).

De senere år er der endvidere kommet mere fokus på den mulige betydning af inflammatoriske mediatorer for udviklingen af det aldersrelaterede muskeltab (Roubenoff 2003) Det skyldes bl.a. at plasma niveauet af TNF-alfa (Tumor Necrosis Factor α) andre proinflammatoriske cytokiner (IL-6, IL-1) og markører for inflammation (CRP, C-reactive protein) er vist at være øget ved stigende alder og ikke mindst at der er fundet at være korrelation mellem højt serum niveau af pro-inflammatoriske cytokiner og lav muskelstyrke (Visser et al. 2002). Det er dog endnu ikke afklaret om det forhøjede TNF-alfa niveau der ses hos ældre personer skyldes aldring, kronisk sygdom eller inaktivitet. Derimod synes det sikkert at TNF-alfa har en katabol effekt på skeletmuskelvæv (Schaap et al. 2006), muligvis som et resultat af apoptoseaktivering og/eller en øget myofibrillær protein nedbrydning (Ground et al. 2008). En mulig forklaring kunne være at de aldersrelaterede molekylære adaptationer associeret med øget TNF-alfa signallering medvirker direkte eller indirekte på udviklingen af sarcopeni (Grounds et al. 2008).

De myogene stamceller (satellitceller) i skeletmuskulaturen vides at spille en central rolle i regenerationen of muskelfibre efter muskelskade, ligesom det er vist at forekomsten og funktionen af satellitcellerne er en afgørende faktor for effektiv muskelnydannelse {Conboy & Rando 2005, Carlson et al 2009}. Nye studier har endvidere vist at aldring nedsætter den regenerative kapacitet af skeletmuskelvæv både hos dyr (Degens & Alway 2003, Siu & Alway 2006) og mennesker

(Carlson et al 2009). Evnen til at opregulere antallet af satellitceller i forbindelse med træning synes dog at være bevaret også hos ældre personer (Mackey et al 2007). Det er således af stor relevans at identificere og udvikle optimale former for intervention med det formål at modvirke og forsinke ovenstående aldringsfaktorer. Samtlige ovenstående faktorer synes at være positivt påvirkelige af fysisk træning (Aagaard et al. 2010). Det er tidligere blevet påvist at styrketræning hos ældre kan lede til nedregulerede inflammationsmarkører (Greiwe et al. 2001). Endvidere har vi set at styrketræning leder til forbedrede muskelmekaniske egenskaber hos ældre personer, svarende til en fysiologisk foryngelse på ~20-30 år hos meget gamle personer (80 år) (Caserotti et al. 2008). Samtidig sker der øgning af muskelmassen og den neuromuskulære funktion inklusive den finmotoriske koordination forbedres (Kryger & Andersen 2002, Suetta et al. 2004, Barry et al. 2005, Hortobagyi et al. 2001). Det er dog ikke tidligere undersøgt i hvilket omfang motionsfodbold kan anvendes til at udløse lignende effekter hos ældre personer.

Gennem tre årtier har en omfattende dansk forskningsindsats kortlagt træningstilstand og præstation hos mandlige og kvindelige elitefodboldspillere (Bangsbo et al. 1991, Mohr et al. 2003, Krustrup et al. 2005) og nylige undersøgelser har tillige undersøgt den fysiske belastning under motionsfodbold og har gennemført træningsstudier med fodboldtræning for utrænede mænd og kvinder i alderen 20-50 år (Krustrup et al. 2009, 2010a,b,c, Andersen et al. 2010a,b). Disse nye resultater viser at motionsfodbold er en særdeles effektiv kombinationstræning der på bare 3 måneder kan lede til forbedredede kardiovaskulære forhold (kondital, hjertefunktion, endotelfunktion, blodtryk, lipidprofil) samt øgning i muskuloskeletal funktion (hypertrofi, styrke, postural balance og knogletæthed). Tillige viser undersøgelser af inaktive danskere at centrale motivationsfaktorer for deltagelse i fysisk aktivitet udgøres af positive sociale oplevelser og tidligere idrætsaktivitet (Skjerk og Ottesen, 2006). En populær og lystbetonet holdsport som fodbold har således et stort potentiale for at tilknytte inaktive mænd til vedvarende motionsaktivitet. Ikke mindre end 90% af alle mænd har på et tidspunkt spillet fodbold og det er velkendt at det sociale element er en vigtig faktor på fodboldhold.

Den træningsmæssige og sundhedsmæssige effekt af motionsfodbold er imidlertid ikke undersøgt hos ældre mænd (65-75 år) ligesom der savnes yderligere videnskabelig dokumentation for de kardio-vaskulære, metabolske og muskulo-skeletale adaptationer ved motionsfodbold.

Hypotese

Det er projektets hovedhypotese at både fodbold- og styrketræning hos ældre mænd (65-75 år) vil lede til, (i) forøget muskelmasse herunder øget areal af explosive type II muskelfibre, (ii) aktivering

af muskulære stamceller (satellit celler), (iii) forbedret muskelmekanisk funktion (øgninger i maximal muskelstyrke, power, kontraktil rate of force development), (iv) forøget knoglemineralisering, (v) forøget neuromuskulær funktion og (vi) forbedret postural balance kontrol. Tillige er hypotesen at fodboldtræning vil lede til (vii) reduceret niveau af inflammations-markører i blodet (viii) forøget kapillarisering samt (ix) forbedret hjertefunktion og kondital.

Forsøgspersoner, herunder inklusions- og eksklusionskriterier

Der vil blive rekrutteret 80 raske mænd i alderen 65-75 år, hvoraf de 20 tilhører en gruppe af raske mænd som har deltaget i livslang fodboldtræning. Disse følges i et år ligesom deltagerne i træningsstudiet. Forsøgsdeltagerne i træningsstudiet må ikke have deltaget i fysisk træning de seneste 10 år. Forsøgspersonerne må ikke have kendte kroniske sygdomme og ej heller være rygere eller have nogen former for misbrug eller tage daglig medicin. Før godkendelse af en forsøgsperson foretages en lægeundersøgelse med måling af blodtryk, hvile-EKG og en glukosebelastningstest. Det er eksklusionskriterier hvis en forsøgsperson under lægeundersøgelsen udviser behandlingskrævende hypertension og hyperglycæmi. Det er tillige et eksklusionskriterium for forsøgspersonerne i træningsforsøget hvis de har et kondital som er lavere end 20 ml/min/kg eller højere end 32,5 ml/min/kg. Antallet af forsøgspersoner er valgt ud fra forventningen om en gennemførelsesprocent på minimum 75%, svarende til minimum 15 deltagere per gruppe, og standardafvigelser for forandringer i main outcomes svarende til ændringer i tilsvarende studier (Krustrup et al. 2009, 2010), hvilket betyder at man ved relevant brug af parametrisk statistik kan opnå signifikante ændringer indenfor hver af træningsgrupperne og evaluere signifikante forskelle grupperne imellem.

Power and sample size beregninger

Forventet detektionsgrænse for ændringer indenfor en gruppe. Parret t-test.

Variabel	SD af ændringer	n=15	n=30
Kondital (ml/min/kg)	2.5	2.0	1.6
Blodtryk (mmHg)	8	6	5
LDL kolesterol (mM)	0.4	0.3	0.2
Muskelmasse (kg)	1.4	1.1	0.7

Power er sat til 0.8 og P til 0.05

Forventet detektionsgrænse for ændringer mellem uafhængige grupper (n=15). One-way ANOVA.

Variabel	SD af ændringer	3 grupper	4 grupper
Kondital (ml/min/kg)	2.5	2.8	3.1
Blodtryk (mmHg)	10	12	13
LDL kolesterol (mM)	0.6	0.7	0.8
Muskelmasse (kg)	1.6	2.0	2.2

Power er sat til 0.8 og P til 0.05

Håndtering af biologisk materiale og personoplysninger

De udtagne muskelbiopsier og blodprøver benyttes til en lang række analyser og der vil derfor kun potentielt være noget overskydende biologisk materiale tilbage efter de planlagte analyser. Eventuelt overskydende biologisk materiale vil herefter blive destrueret. Projektet anmeldes til Datatilsynet. Oplysninger vedrørende forsøgspersonerne beskyttes efter lov om behandling af personoplysninger og lov om patienters retsstilling. Hver enkelt forsøgsperson vil efter projektafviklingen have mulighed for at få adgang til egne data samt gennemsnitsdata for hele gruppen. I den skriftlige deltagerinformation spørges om hvorvidt forsøgsdeltagerne ønsker adgang til egne forsøgsdata. 2 måneder efter projektafslutning. Alle data vil blive anonymiseret.

Design

80 mandlige forsøgspersoner med en alder på 65-75 år rekrutteres til forsøget. I alt 20 af deltagerne har været fysisk aktive som fodboldspillere hele deres voksenliv (seneste 40 år), mens de resterende 60 deltagere er raske, utrænede forsøgspersoner som ikke har dyrket regelmæssig motion de seneste 10 år. De 60 utrænede forsøgspersoner randomiseres (stratificeret for kondital og muskelstyrke) til én ud af tre forsøgsgrupper: En **fodboldtræningsgruppe** (n=20), en **styrketræningsgruppe** (n=20) og en **inaktiv kontrolgruppe** (n=20). De to træningsgrupper træner først 2x45 min per uge (de første 4 uger), dernæst 2x1 time per uge (de næste 4 uger) og herefter 3x1 time per uge i resten af interventionsperioden. Fodboldgruppen træner udendørs på græs og kunstgræs ved 5 mod 5 og 7 mod 7 til to mål. Styrketræningsgruppe træner indendørs med tung styrketræning (6-10 RM, 1-3 sæt) fortrinsvis for benene.

Metoder

Der gennemføres i alt 3 testrunder (0, 3 og 12 måneder). Her gennemføres lægeundersøgelser med målinger af ekg og liggende blodtryk. De tre testrunder er beskrevet i detaljer nedenfor. I forbindelse med hver af testrunderne gennemføres tests over 4 dage af 2-5 timers varighed. Testdagene foregår med minimum 48 timers mellemrum. Deltagerne må ikke have lavet anstrengende fysisk arbejde dagen før en testdag. De må ikke have drukket alkohol dagen op til testdagen og må ikke drikke te, kaffe eller ryge på selve testdagen. I 24 timer før testdagene indtages en standardiseret kulhydratrig kost.

Der udtages hvileblodprøver (10 ml) i faste fra perifer vene ved indlæggelse af venflon i albuebøjningen og ifm glukosebelastningstesten og cykeltesten (i alt 50 ml; testdag 1 og 2, se

nedenfor). Tillige udtages - for deltagerne i de to træningsgrupper - 10 blodprøver i forbindelse med et træningspas (40 ml i alt). For kontrolpersonerne udtages altså i alt 180 ml blod gennem hele den 1 år lange projektperiode, mens der for deltagerne i træningsgruppen udtages i alt 220 ml blod.

I forbindelse med hver af de tre testrunder (0, 3 og 12 måneder) udtages en tillige en muskelprøve fra lårmuskulaturen som vejer 0,1 g. For deltagerne i de to træningsgrupper udtages også en muskelprøve umiddelbart efter en enkelt træningssession. For kontrolpersonerne udtages altså i alt 3 muskelprøver og for deltagerne i træningsgruppen udtages i alt 4 muskelprøver, svarende til mindre en et halvt gram muskelvæv.

Blodprøver - mhp måling af kolesteroltal, blodsukker, insulin, katekolaminer, højsensitiv CRP, samt TNF-alfa og IGF-1.

Muskelbiopsier - Muskelprøverne udtages fra m. vastus lateralis under sterile forhold. Under lokalbedøvelse foretager projektets læge et snit i huden på ca. 0,5 cm hvorefter muskelprøven udtages med en biopsinål. Muskelprøverne benyttes til histokemisk analyse af fibertyper, fiberarealer og kapillærer samt bestemmelse af satellit celle aktivering med immunohistokemisk farvning for N-CAM [neural cell adhesion molecule) og Pax-7 (paired-box transcription factor)]: (Crameri et al. 2007). Tillige undersøges det om der sker ændringer i muskelfibernes genexpression af myostatin, MGF, IGF-1, TNF-alfa, atrogin, ubiquitin og calpainer, hvor vævsanalyserne foretages ved anvendelse af realtime-PCR teknik og LDA kort.

Pulsmålinger I forbindelse med alle træningssessioner foretages pulsmålinger og ved udvalgte træningssessioner efter 1, 6, 12 og 50 uger bestemmes den fysiske aktivitetsprofil ved videofilmning og accelerometermålinger, samt selvoplevet belastningsgrad. I forbindelse med en træningssession efter 12 uger udtages tillige blodprøver og en muskelbiopsi (se nedenfor).

DXA-scanning - Der foretages en helkrops DXA-scanning til bestemmelse af fedtprocent, muskelmasse og knoglemineralisering, samt en lokal DXA-scanning til måling af knoglemasse og –knoglemineralisering af lårben og lårbenshals.

Glukosebelastningstest - Der gennemføres en oral glukosebelastningstest over 2 timer. Der udtages blodprøver til måling af blodsukker og –insulin efter 0 min, 15 min, 30 min, 1 time og 2 timer efter sukkerindtagelsen.

Perifer arterietonometri - Karfunktion måles siddende i hvile ved hjælp af p*erifer arterietonometri* (PAT) metoden. PAT måler pulsbølgeamplituden under reaktiv hyperæmi ved hjælp af en fingerpletysmograf. Det i protokollen anvendte Endo-PAT-system udmærker sig ved, at der måles med en fingerprobe på hver arm, dvs. forsøgsdeltageren er sin egen kontrol. Der er ingen kendte risici ved metoden.

Ekkokardiografi - Efterfølgende måles hjertefunktion ved avanceret vævsdoppler ekkokardiografi til undersøgelse af muskelvævets kontraktions- og relaxationsmønstre. Der anvendes 2-D strain analyser, som tillader vinkel uafhængige analyser af såvel longitudinelle som radielle kontraktions mønstre og i tillæg vurderes circumferentielle bevægemønstre, og dermed selve twist bidraget til den globale myokardiefunktion. Undersøgelser foretages af sammenhæng mellem radial og longitudinel kontraktion, relation mellem twist (circumferentiel rotation ved apex og basis, relation mellem twist og diastolisk funktion, relation mellem twist, diastolisk funktion og atriedimension og ændringer under belastning (Amundsen et al. 2006, Poulsen et al. 2007, Notomi et al. 2008). Målingerne foregår mens forsøgsdeltagen liggende på siden. Der er ingen kendte risici ved metoden.

Cykeltest - Der gennemføres en cykeltest til bestemmelse af maximal iltoptagelseshastighed (kondital), fedtforbrændingsevne ved sub-maximalt arbejde og arbejdsevne under et standardiseret maksimalt arbejde. Cykel testen består af 6 minutters sub-maximalt arbejde efterfulgt af 4 minutters hvile og en progressiv maksimalttest. Den progressive maksimaltest starter med 4 minutters arbejde på en belastning på 40 W, hvorefter belastningen hæves med 20 W hvert minut indtil udmattelse. Under protokollen udtages i alt 4 blodprøver fra en armvene, henholdsvis før og efter hver af de to arbejdsperioder, til måling af mælkesyre, sukker, FFA, kalium og ammoniak. Tillige måles puls med et Polar pulsbælte og iltoptagelse, ventilation og respiratorisk udvekslingskvotient (RER) måles med on-line gasanalysator, der måler på ud- og indåndingsluften (Med Graphics, USA).

Muskelfunktion - Der måles postural balance, maximal hoppehøjde, maximal powerudvikling og styrke i benmuskulaturen. Efter en standardiseret opvarmning på cykel måles postural balance og maximal hoppehøjde og power-udvikling på en kraftplatform (Jakobsen et al. 2011, Caserotti et al, 2008). Personernes balance evne måles tillige på en 5 cm høj balancebom, hvor antallet af berøringer med jorden indenfor 1 min noteres (Jakobsen et al. 2011). Dernæst måles benmuskulaturens styrke ved en række tests udført i et isokinetisk dynamometer (KinCom)

(Aagaard et al. 2002). Der måles styrke i for- og baglår både ved bevægelse og i en fastlåst stilling. Tillige måles reaktionstid ved pludselig rygbelastning (Pedersen et al. 2009).

Sprintevne og intervalarbejdsevne - Efter en standardiseret opvarmning udføres en 20-m sprint test. Hver deltager får 2 forsøg i sprinttesten. Den bedste præstation noteres og benyttes som testresultat. Derefter gennemføres en Yo-Yo Interval Udholdenhedstest – niveau 1.

Testdag 1:

På Institut for Idræt, Københavns Universitet, Universitetsparken 13, 2. sal, foretages lægeundersøgelse med måling EKG og blodtryk i fastende hvile. Der udtages en blodprøve fra en armvene med henblik på måling af blodsukker, insulin, kolesterol, proinflammatoriske cytokiner og triglycerider. Der foretages tillige en helkrops DXA-scanning til bestemmelse af fedtprocent, muskelmasse og knoglemineralisering, samt en lokal DXA-scanning af begge lår til bestemmelse af knoglemasse og knoglemineralisering af lårben og lårbenshals. Herefter gennemføres en oral glukosebelastningstest over 2 timer. Der udtages blodprøver til måling af blodsukker og –insulin efter 0 min, 15 min, 30 min, 1 time og 2 timer efter sukkerindtagelsen. Efter indtagelse af morgenmad udtages en muskelprøve fra lårmuskulaturen (se detaljeret beskrivelse nedenfor) med henblik på måling af musklens indhold af sukker, samt fiberstørrelse og fibertype sammensætning. Testdagen varer 4 timer.

Testdag 2:

På Institut for Idræt, Københavns Universitet, Universitetsparken 13, 2. sal, gennemføres non-invasive målinger af kar- og hjertefunktion, samt en cykeltest til bestemmelse af kondital og arbejdsevne. Dagen op til testen indtages en standardiseret kulhydratrig kost og om morgenen indtages standardiseret morgenmad. I siddende hvile måles karfunktion ved p*erifer arterietonometri* (PAT) metoden. PAT måler pulsbølgeamplituden under reaktiv hyperæmi ved hjælp af en fingerpletysmograf. Det i protokollen anvendte Endo-PAT-system udmærker sig ved, at der måles med en fingerprobe på hver arm, dvs. forsøgsdeltageren er sin egen kontrol. Der er ingen kendte risici ved metoden. Efterfølgende måles hjertefunktion ved avanceret vævsdoppler ekkokardiografi til undersøgelse af muskelvævets kontraktions- og relaxationsmønstre. Der anvendes 2-D strain analyser, som tillader vinkel uafhængige analyser af såvel longitudinelle som radielle kontraktions mønstre og i tillæg vurderes circumferentielle bevægemønstre, og dermed selve twist bidraget til den globale myokardiefunktion. Undersøgelser foretages af sammenhæng mellem radial og longitudinel kontraktion, relation mellem twist (circumferentiel rotation ved apex og basis, relation

mellem twist og diastolisk funktion, relation mellem twist, diastolisk funktion og atriedimension og ændringer under belastning (Amundsen et al. 2006, Poulsen et al. 2007, Notomi et al. 2008). Målingerne foregår mens forsøgsdeltagen liggende på siden.

Herefter gennemføres en cykeltest til bestemmelse af maximal iltoptagelseshastighed (kondital), fedtforbrændingsevne ved sub-maximalt arbejde og arbejdsevne under et standardiseret maksimalt arbejde. Cykel testen består af 6 minutters sub-maximalt arbejde efterfulgt af 4 minutters hvile og en progressiv maksimalttest. Den progressive maksimaltest starter med 4 minutters arbejde på en belastning på 40 W, hvorefter belastningen hæves med 20 W hvert minut indtil udmattelse. Under protokollen udtages i alt 4 blodprøver fra en armvene, henholdsvis før og efter hver af de to arbejdsperioder, til måling af mælkesyre, sukker, FFA, kalium og ammoniak. Tillige måles puls med et Polar pulsbælte og iltoptagelse, ventilation og respiratorisk udvekslingskvotient (RER) måles med on-line gasanalysator, der måler på ud- og indåndingsluften (Med Graphics, USA). Testdag 2 varer ca. 5 timer.

Testdag 3:

På Bispebjerg Hospital, Bispebjerg Bakke 23, Bygning 8, 2400 København NV, måles balance, hoppehøjde, styrke samt reaktionstid ved rygbelastning. Personerne har spist en standardiseret kulhydratrig kost dagen inden testen og et let måltid 1½-2 timer før testen. Der måles postural balance, maximal hoppehøjde, maximal powerudvikling og styrke i benmuskulaturen. Efter en standardiseret opvarmning på cykel måles postural balance og maximal hoppehøjde og power-udvikling på en kraftplatform (Jakobsen et al. 2011, Caserotti et al, 2008). Personernes balance evne måles tillige på en 5 cm høj balancebom, hvor antallet af berøringer med jorden indenfor 1 min noteres (Jakobsen et al. 2011). Dernæst måles benmuskulaturens styrke ved en række tests udført i et isokinetisk dynamometer (KinCom) (Aagaard et al. 2002). Der måles styrke i for- og baglår både ved bevægelse og i en fastlåst stilling. Tillige måles reaktionstid ved pludselig rygbelastning (Pedersen et al. 2009). Denne testdag varer 2 timer.

Testdag 4:

På Institut for Idræt, Nørre Allé 51, gennemføres testning af sprintevne og intervalarbejdsevne. Denne testdag består af to præstationstest som foregår indendørs på en håndholdbane. Efter en standardiseret opvarmning udføres en 20-m sprint test. Hver deltager får 2 forsøg i sprinttesten. Den bedste præstation noteres og benyttes som testresultat. Derefter gennemføres en Yo-Yo Interval Udholdenhedstest – niveau 1. I forbindelse med denne test løbes 2x20 meter frem og tilbage mellem toppemarkeringer. Efter hver tur holdes 5 sekunders pause. Hastigheden under løbeturene

justeres gradvist opad efter bip-signaler på et bånd eller CD. Når man første gang ikke når tilbage til målstregen i tide, får man en advarsel og anden gang er testen slut. Der er en læge til stede under disse tests. Den tilbagelagte distance noteres som testresultat. Testdag 1 varer ca. 2 timer.

Målinger under træning

For deltagerne i de to træningsgrupper vil der i forbindelse med hver træningssession blive foretaget måling af pulsfrekvens ved hjælp af et Polar pulsur. Ved udvalgte træningssessioner efter 1, 6, 12 og 50 uger vil tillige blive udleveret spørgeskemaer om selvoplevet belastningsgrad og der vil blive foretaget accelerometermålinger samt videofilmninger med henblik på gennemførelse af en computerbaseret analyse af bevægelsesmønster. I forbindelse med træningssessionen efter 12 ugers træning vil blive udtaget i alt 10 blodprøver fra en arm vene og umiddelbart efter træningssessionen vil blive udtaget en muskelprøve fra lårmuskulaturen.

Træningsprotokol

Styrke træning

Træningen omfatter forskellige bentræningsøvelser (benpres, knæ ekstension, hamstring curl) som i de første 4 uger udføres med belastninger svarende til 12-16 RM (12 RM er den vægt der kan løftes maximalt 12 gange), derefter gøres belastningerne tungere svarende til 10-12 RM i ugerne 5-8, og til 8-10 RM ugerne 9-12. Vi har tidligere gennemført styrketræningsstudier med denne type af træningsøvelser for ældre personer (60-85 år), hvor der ikke er iagttaget nogle risici eller bivirkninger i forbindelse med den gennemførte træning (Suetta et al. 2004a,b; Caserotti et al. 2008).

Fodboldtræning

I lighed med nylige undersøgelser af 30-55 årige utrænede mænd bliver fodboldtræningen gennemført som småspil på kunstgræs eller almindeligt græs. Småspillene gennemføres ved 5-, 6- eller 7-a-side til små mål (kegler eller 2x5 m mål). Der foretages let opvarmning med dribling og boldøvelser i 10 minutter, hvorefter der spilles 4x12 minutter adskilt af 3 minutters pauseperioder med tid til at drikke vand og snakke taktik (Krustrup et al. 2010, Andersen et al. 2010).

Det er velkendt at der er en markant højere skadesrisiko ved fodboldkampe (5-10 gange højere) end ved fodboldtræning (Krustrup et al. 2010). Alligevel skal det understreges at muskelforstrækninger (i bl.a. baglår og forlår) og vridskader (i bl.a. ankler og knæ) også kan opstå under træning på små mål.

Bivirkninger, risici og ulemper for forsøgspersonerne

I forbindelse med DXA-scanningerne udsendes svage røntgenstråler, hvorved kroppens sammensætning af forskellige vævstyper måles. Der udsendes 0,0006 mSv ved helkrops-DXA og 0,005 mSv ved lokal DXA-scanning af lårben og lårbenshals, i alt 0,0056 mSv. Det svarer til en dosis, der giver en forøget kræftrisiko på 0,001%, hvilket må anses for at være en minimal risiko. Den stråledosis forsøgspersonerne får er mindre end ved de fleste røntgenundersøgelser og svarer til 7 dages baggrundsstråling. DXA-scanningerne gør ikke ondt og er ikke forbundet med nogen form for ubehag. I forbindelse med lægeundersøgelsen, cykeltesten og glukosebelastningstestene tages blodprøver fra en armvene. Der indlægges et kateter i en vene i albuebøjningen. I forbindelse med fjernelse af kateterne kan der opstå en mindre blødning, der giver misfarvning af huden. For at minimere denne gene vil der blive komprimeret efter udtagelsen (let tryk på stedet). Brug af katetre er benyttet rutinemæssigt på Institut for Idræt og Bispebjerg Hospital, og er brugt uden betydende komplikationer i mere end 30 år. Ved udtagning af muskelbiopsier lægges først en lokalbedøvelse med lidokain af huden, hvorefter der laves et snit i huden på ca. 0,5 cm. Herefter udtages muskelbiopsierne, der hver vejer ca. 0,1 gram. Udtagning af muskelbiopsier er en rutineprocedure, der dagligt udføres på Institut for Idræt, Københavns Universitet, og Bispebjerg Hospital, og som uden komplikationer også foretages hos ældre individer (Suetta et al. 2008, Aagaard et al. 2007). Der kan føles et ubehag og i nogen tilfælde smerte, i det øjeblik biopsien tages og ved udtagning af nåle-biopsier er der en meget lile risiko for at sensoriske nerver beskadiges lokalt. Dette kan sjældent betyde at følesansen i et mindre område omkring indstiksstedet (på størrelse med en 5-krone) er nedsat i op til et år efter forsøget. I meget sjældne tilfælde (>1/5000) kan der ske beskadigelse en lokal motorisk nerve. I så fald kan evnen til at aktivere en lille del af musklen mistes, men totalt set vil muskelfunktion for den undersøgte muskelgruppe være helt upåvirket. Der kan tillige være uforudsigelige risici og belastninger knyttet til deltagelse i videnskabelige forsøg. Desuden er der potentiel risiko for infektion i insertionsstederne, hvor muskelbiopsierne er taget igennem. Risikoen for infektion modvirkes primært ved at prøverne udtages under sterile forhold samt grundig instruktion til deltagerne om behandling af biopsi-insertionsstederne. Udtagelse af muskelbiopsier er rutine på forsøgslaboratoriet, og er forløbet uden betydende komplikationer i mere end 25 år. Der kan udføres arbejde/fysisk aktivitet straks efter muskelbiopsier. Der vil dog være ømhed i ca. 2-4 dage efter biopsi udtagelsen.

Initiering af projektet og eksterne projektmidler

Projektet er initieret af Peter Krustrup, PhD og lektor på Institut for Idræt, Københavns Universitet og Charlotte Suetta, PhD, læge på Klinisk Fysiologisk og Nuklear-Medicinsk Afdeling, Bispebjerg Hospital. Projektet har modtaget støtte fra Kulturministeriets Udvalg for Idrætsforskning med kr.

300.000.- og fra forskningsenheden i det internationale fodboldforbund FIFA (F-MARC) med kr. 850.000.- Der er tillige søgt økonomisk støtte hos EU. Projektmidlerne er udbetalt til en forskningskonto (konto 31964) på Institut for Idræt, Københavns Universitet og bruges dels til indkøb af udstyr, til blod- og muskelanalyser, samt til aflønning af forskningsassistent. Der er tillige afsat midler til overhead. Der er ingen i projektgruppen som har tilknytning til private virksomheder med interesser i projektet.

Der vil blive udbetalt kr. 2500.- i vederlag til hver af forsøgspersonerne for deltagelse i projektet. Udbetaling foregår når man som forsøgsperson har afsluttet sin deltagelse. Beløbet er skattepligtigt.
Når man tager i betragtning at forsøgsdeltagerne gennemfører 12 testdage og deltager i op til 140 træningspas, samt at der udtages 4 muskelbiopsier fordelt over 3 dage og armvene-blodprøver fordelt over i de tre testningsrunder, er beløbet tilpas af beskeden størrelse til at der ikke sker en utilbørlig påvirkning af forsøgspersonerne.

Rekruttering af forsøgspersoner

Forsøgspersonerne rekrutteres primært via annoncer i lokalaviser (primært Østerbro, Nørrebro) og gratisaviser (Urban og MetroExpress). De trænede fodboldspillere rekrutteres endvidere gennem fodboldklubber i hovedstadsområdet. Opslags- og annoncetekst er vedlagt som bilag.

Formidling af forsøgsresultater

Det er planen at projektets resultater skal formidles bredt og indgå i den samfundsmæssige debat. I sommerperioden 2012 forventes organiseret en pressekonference om resultaterne, med henblik på en mediebaseret offentliggørelse. Undersøgelsens resultater præsenteres tillige ved videnskabelige kongresser sommeren og efteråret 2012 og ved udgivelsen af minimum to videnskabelig artikler i 2012. Det er tillige planlagt at resultaterne skal offentliggøres i forbindelse med en række sundhedskonferencer, hvor fremtidens kommunalt baserede sundhedsfremmende indsats er til debat. Såvel positive som negative resultater vil blive offentliggjort.

Forsikringsforhold

Samtlige deltagere i forsøget er omfattet af patientskadeforsikringen indenfor H:S.

Etiske overvejelser

Henholdsvis fodboldtræning og styrketræning i 12 måneder anses for at være træningsforbedrende og generelt sundhedsfremmende for de rekrutterede forsøgspersoner. Ved den indledende

lægeundersøgelse foretages måling af blodtryk og EKG samt glukosebelastningstest for at fastslå om forsøgspersonen kan inkluderes i forsøget. Hvis ikke, sendes forsøgspersonen til videre udredning, primært hos egen læge. Hvad angår forsøgspersonerne i kontrolgruppen skal de lave det de plejer og gives gennem deltagelsen et lægecheck og andre oplysninger af personlig interesse. Kun raske forsøgspersoner kan indgå i kontrolgruppen – personer med hypertension, hyperglykæmi og andre sygdomme sendes videre til specifik behandling. Der udbetales et beskedent vederlag for svie og smerte, som ikke anses for at bevirke en utilbørlig påvirkning af forsøgspersonerne. I forbindelse med forsøget udtages i alt 4 muskelprøver fra lårmuskulaturen. Udtagelse af muskelbiopsier foregår under lokalbedøvelse af det ovenliggende hudområde. For forsøgspersonerne kan der føles et ubehageligt tryk i musklen ved udtagningen, samt ømhed 2-3 dage efter udtagningen, indimellem svarende til et ”trælår”. Desuden efterlader biopsitagningen små ar på huden, der typisk er næsten usynlige 1-2 år efter forsøget. Blodprøver (højest 100 ml blod per forsøgsrunde svarende til ca. 2% af den samlede blodmængde) og muskelbiopsier bruges til at afklare projektets problemstillinger og anses ikke som noget etisk problem for en gruppe af raske 65-75 årige. Forsøget udføres i fuld overensstemmelse med Helsinki–deklarationen. Forsøget anmeldes til Datatilsynet. Oplysninger vedrørende forsøgspersonerne beskyttes efter lov om behandling af personoplysninger og lov om patienters retsstilling. Eventuelt overskydende biologisk materiale vil blive destrueret. Både positive og negative resultater vil blive offentliggjort.

Afgivelse af mundtlig information og indhentelse af samtykke

Efter at have udvist interesse modtager potentielle forsøgspersoner det skriftlige materiale (den vedlagte forsøgspersonsinformation og folderen ”før du beslutter dig”). Ugen efter indkaldes forsøgspersonerne til samtale, hvor projektgruppens medlemmer supplere den skriftlige information med mundtlig information om baggrunden for forsøget, formålet og forsøgsprotokollen. Herefter vil den tilknyttede projektlæge i forbindelse med en personlig samtale give mundtlig information om de anvendte metoder samt eventuelle risici og bivirkninger. Forsøgspersonerne gøres opmærksom på, at de kan medbringe en bisidder til dette møde. Dette møde foregår uforstyrret på et egnet kontor. Det endelige tilsagn og underskrift på samtykkeerklæringen indhentes efter minimum 2-3 dages betænkningstid. Det understreges, at man når som helst kan trække sig ud af forsøget.

REFERENCER

Amundsen BH, Helle-Valle T, Edvardsen T, Torp H, Crosby J, Lyseggen E, Støylen A, Ihlen H, Lima JA, Smiseth OA, Slørdahl SA (2006). Noninvasive myocardial strain measurement by speckle tracking echocardiography: validation against sonomicrometry and tagged magnetic resonance imaging. J Am Coll Cardiol 47:789-93.

Aagaard P, Magnusson SP, Larsson B, Kjær M, Krustrup P (2007). Mechanical muscle function, morphology and fibertype in life-long trained elderly. Med Sci Sports Exerc 39: 1989–1996.

Aagaard P, Simonsen EB, Andersen JL, Magnusson SP, Dyhre-Poulsen P (2002). Training induced increases in muscle contractile Rate of Force Development and neural drive. J Appl Physiol 93: 1318-1326.

Aagaard P, Suetta C, Caserotti P, Magnusson SP, Kjær M (2010). Role of the nervous system in sarcopenia and muscle atrophy with aging - strength training as a countermeasure. Scand J Med Sci Sports 20: 49-64.

Andersen LJ, Hansen PR, Søgaard P, Madsen JK, Bech J, Krustrup P. (2010). Improvement of systolic and diastolic heart function after physical training in sedentary women. Scand J Med Sci Sports20, suppl 1: 50-57.

Andersen LJ, Randers MB, Westh K, Martone D, Riis Hansen P, Junge A, Dvorak J, Bangsbo J, Krustrup P (2010). Football as treatment of hypertension for untrained 30-55 year old men – a prospective randomised study. Scand J Med Sci Sports 20, suppl 1: 98-102.

Caserotti P, Aagaard P, Larsen JB, Puggaard P. Explosive heavy-resistance training in old and very old adults:changes in rapid muscle force, strength and power. Scand. J. Med. Sci. Sports 18, 773-782, 2008

Helge EW, Aagaard P, Jakobsen MD, Sundstrup E, Randers MB, Karlsson MK, Krustrup P. (2010). Recreational football training decreases risk factors for bone fractures in untrained premenopausal women. Scand J Med Sci Sports 20, suppl 1: 31-39.

Krustrup P, Nielsen JJ, Krustrup B, Christensen JF, Pedersen H, Randers MB, Aagaard P, Petersen AM, Nybo L, Bangsbo J (2009). Recreational soccer is an effective health promoting activity for untrained men. Br J Sports Med. 43(11): 825-831.

Krustrup P, Aagaard P, Nybo L, Petersen J, Mohr M, Bangsbo J (2010). Recreational football as a health promoting activity: a topical review. Scand J Med Sci Sports 20, suppl 1: 1-13.

Krustrup P, Christensen JF, Randers MB, Pedersen H, Sundstrup E, Jakobsen MD, Krustrup BR, Nielsen JJ, Suetta C, Nybo L, Bangsbo J (2010). Muscle adaptations and performance enhancements of soccer training for untrained men. Eur J Appl Physiol. 108(6):1247-1258.

Krustrup P, Hansen PR, Randers MB, Nybo L, Martone D, Andersen LJ, Bune LT, Junge A, Bangsbo J (2010). Beneficial effects of recreational football on the cardiovascular risk profile in untrained premenopausal women. Scand J Med Sci Sports 20, suppl 1: 40-49.

Notomi Y, Popovic ZB, Yamada H, Wallick DW, Martin MG, Oryszak SJ, Shiota T, Greenberg NL, Thomas JD (2008). Ventricular untwisting: a temporal link between left ventricular relaxation and suction. Am J Physiol Heart Circ Physiol 4:H505-13.

Pedersen MT, Randers MB, Skotte JH, Krustrup P. (2009). Recreational soccer can improve the reflex response to sudden trunk loading among untrained women. J Strength Cond Res. 2009 23(9):2621-6.

Poulsen SH, Hjortshøj S, Korup E, Poenitz V, Espersen G, Sogaard P, Suder P, Egeblad H, Østergaard Kristensen B (2007). Strain rate and tissue tracking imaging in quantitation of left ventricular systolic function in endurance and strength athletes. Scand J Med Sci Sports 2:148-55.

Randers MB, Nybo L, Petersen J, Nielsen JJ, Christiansen L, Bendiksen M, Brito J, Bangsbo J, Krustrup P. (2010). Activity profile and physiological response to football training for untrained males and females, elderly and youngsters: influence of the number of players. Scand J Med Sci Sports. 20, suppl 1: 14-23.

Rasmussen U., P. Krustrup, M. Kjær & H.N. Rasmussen (2003). Human skeletal muscle mitochondrial metabolism in youth and senescence: no signs of functional changes in ATP formation and mitochondrial oxidative capacity. Pflügers Arch. - Eur. J. Physiol. 446(1): 270-278.

Rasmussen U., P. Krustrup, M. Kjær & H.N. Rasmussen (2003). Experimental evidence against the mitochondrial theory of aging. A study of isolated human skeletal muscle mitochondria. Exp. J. Geront. 38(7): 726-735.

Suetta C, Aagaard P, Rosted A, Jakobsen AK, Duus B, Kjaer M, Magnusson SP. Training-induced changes in muscle CSA, muscle strength, EMG and rate of force development in elderly subjects after long-term unilateral disuse. J. Appl. Physiol. 97, 1954-1961, 2004

Suetta C, Andersen JL, Dalgas U, Berget J, Koskinen S, Aagaard P, Magnusson SP, Kjaer M (2008). Resistance training induces qualitative changes in muscle morphology, muscle architecture and muscle function in elderly postoperative patients. J Appl Physiol 105: 180-186.

Suetta C, Magnusson SP, Rosted A, Aagaard P, Jakobsen AK, Larsen LH, Duus B, Kjaer M. Resistance training in the early post-operative phase reduces hospitalization and leads to muscle hypertrophy in elderly hip surgery patients – a controlled randomized study. J. Am. Geriatr. Soc. 52, 2016-2022, 2004
