# Supplementary material for: The Effects of 52 Weeks of Soccer or Resistance Training on Body Composition and Muscle Function in +65-Year-Old Healthy Males – A Randomized Controlled Trial
Source: PLoS One. 2016 Feb 17;11(2):e0148236. doi: 10.1371/journal.pone.0148236 (PMC4757560; doi:10.1371/journal.pone.0148236)
Supplement: S2 Protocol — (PDF) [file pone.0148236.s003.pdf]

København, 1. april 2011

(Shortened English transcript)
Application to:
De Videnskabsetiske Komitéer for Region Hovedstaden
Regionsgården, Kongens Vænge 2, 3400 Hillerød

Soccer and resistance training as physical activity interventions in 65-75-year-old men: influence on muscle mass, neuromuscular function and cardiovascular risk factors.

Purpose

The purpose of the study is to investigate and compare the musculoskeletal, neuromuscular, and cardiovascular effects of soccer and resistance training, respectively, in 65-75-year-old healthy untrained men. Muscle fibre size, satellite cells, muscle strength, postural balance, bone density, oxygen uptake, blood pressure, blood cholesterol and triglycerides, insulin sensitivity and body fat is investigated in 60 old men participating in a randomized clinical trial lasting 12 months.. Additional 17 subjects are recruited from local football clubs and have been active players for the last 40 years.

Hypotheses

Both soccer and resistance training in 65-75-year-old healthy untrained men leads to (i) increased muscle mass including increased Type IIx muscle fibre area, (ii) activation of muscular stem cells, (iii) improved muscle mechanic function, (iv), increased bone mineralization, (v) improved neuromuscular function, (vi) improved postural balance. Moreover, soccer training leads to, (vii) reduced inflammation, (viii) increased capillarization, (ix) improved cardiac function and oxygen uptake.

Subjects

Eighty (60) 65-75-year-old healthy untrained men are recruited and equally randomized into a soccer, resistance and a control group, respectively. Exclusion criteria are symptoms or history of cardiovascular disease, hyperglycaemia, diagnosed hypertension, or Vo2max below or above 20 ml/min/kg or 32.5 ml/min/kg, respectively. The participant may not have been physically active for the last 10 years prior to enrollment in the study. The number of participants is chosen based on an expected completion of at least 75% corresponding (n=15), standard deviation equal to expected

changes found in other studies (Krustrup et al. 2009, 2010a+b). As such, relevant application of parametric statistics will detect significant changes within and between the allocated groups.

NOTE: Due to limited response to the study advertisements in the local news paers, a reduced number of participants were included in the study.

Krustrup P, Nielsen JJ, Krustrup B, Christensen JF, Pedersen H, Randers MB, Aagaard P, Petersen AM, Nybo L, Bangsbo J (2009). Recreational soccer is an effective health promoting activity for untrained men. Br J Sports Med. 43(11): 825-831.

Krustrup P, Aagaard P, Nybo L, Petersen J, Mohr M, Bangsbo J (2010a). Recreational football as a health promoting activity: a topical review. Scand J Med Sci Sports 20, suppl 1: 1-13.

Krustrup P, Christensen JF, Randers MB, Pedersen H, Sundstrup E, Jakobsen MD, Krustrup BR, Nielsen JJ, Suetta C, Nybo L, Bangsbo J (2010b). Muscle adaptations and performance enhancements of soccer training for untrained men. Eur J Appl Physiol. 108(6):1247-1258.

Power and sample size calculations

Expected detection limits for changes within a group. Paired t-test.

Variable	SD of changes	n=15	n=30
VO2max (ml/min/kg)	2.5	2.0	1.6
BP(mmHg)	8	6	5
LDL cholesterol (mM)	0.4	0.3	0.2
Muscle mass (kg)	1.4	1.1	0.7

Power = 0.8 and P = 0.05

Expected detection limits for changes between unrelated groups. One-way ANOVA.

Variable	SD of changes	3 grupper	4 grupper
VO2max (ml/min/kg)	2.5	2.8	3.1
BP(mmHg)	10	12	13
LDL cholesterol (mM)	0.6	0.7	0.8
Muscle mass (kg)	1.6	2.0	2.2

Power = 0.8 and P = 0.05

Design and training protocol

The trainings groups exercise for 2x45 min per week (week 0-4), 2x1 h per week (week 5-8) and 3x1 h per week for the rest of the intervention period. The soccer group performs small-sided games 5-, 6- or 7-a-side. After a low intensity warm-up 4 games lasting 12 min are conducted separated by short rest periods (3 min). The resistance training group peforms a 5 min of low intensity warm-up followed by heavy resistance training primarily for the legs (6-10 RM, 1-3 set). The resistance training includes legpres, knee extension, hamstring curl. At the end of each training session 5 min of core training (crunches, hip extension, side bends, diagonal lifts, and trunk rotation) is performed. The control group continues a sedentary lifestyle.

NOTE: For practical reasons, the training protocol was slightly changed to a 1-h training session twice per week for 16 weeks, and a 1-h training session three times per week for the following 36 weeks. During weeks 0–4, weeks 5–8, weeks 9–12, and weeks 13–16 strength training intensity was progressed as 16–20 reps (16–20 RM), 12 reps (12 RM), 10 reps (10 RM), and 8 reps (8 RM), respectively,

Testing session and methods

In total of three testing session is conducted throughout the study period (0,3 and 12 months). Medical examination is performed at baseline including measurements of ECG and blood pressure. The subjects must sustain from physical activity the day before a test session in addition to ingesting a standardized CHO-rich diet the evening before the test day. The subjects are not allowed to ingest alcohol, coffee or the on the day of the testing. The test protocol is conducted over four separate days separated by at least 48-h.

Blood samples and muscle biopsies are collected at rest during each test session (n=3). In addition, the subjects in the training groups will have one additional muscle biopsy taken during a selected training session.

NOTE: Testing session were performed at 0, 4 and 12 months.

Outcomes

Primary outcomes:

Primary endpoints will be changes in cardiac and vascular structure and function measured by advanced echocardiography (tissue Doppler and speckle tracking imaging), and peripheral arterial tonometry (Endo-PAT device).

Secondary outcomes:

Secondary endpoints will be evaluation of potential changes in VO2 max, blood pressure, muscle capillaries, lipid profile, circulating catecholamines, body composition, and insulin resistance.

Measurements

Blood analysis - Measurements af blood cholesterol, triglycerides, insulin, blood sucker, chatecholamines, HS-CRP, TNF-alfa og IGF-1.

NOTE: Inflammatory markers remains to be analyzed and will be published elsewhere.

Muscle biopsy analysis – muscle tissue is analysed immunohistochemical for fibre types fibre type and distribution, capillarization in addition to analysis for Pax-7 (paired-box transcription factor). Also, changes in the genexpression for myostatin MGF, IGF-1, TNF-alfa, atrogin, ubiquitin and calpaines is evaluated by realtime-PCR tegnique and LDA card.

NOTE: Gene analysis remains to be analyzed and will be published elsewhere. Standard Western Blot analysis were applied to evaluate expression of selected proteins.

Heart rate measurements: During selected training session heart rate is measured throughout the session to determine physiological load

NOTE: Published in Andersen TR, Schmidt JF, Nielsen JJ, Randers MB, Sundstrup E, Jakobsen MD, Andersen LL, Suetta C, Aagaard P, Bangsbo J, Krustrup P. Effect of football or strength training on functional ability and physical performance in untrained old men. Scand J Med Sci Sports. 2014 Aug;24 Suppl 1:76-85. doi: 10.1111/sms.12245. Epub 2014 Jun 5.

DXA-scanning - Whole body and regional fat mass and lean mass is determined by whole body Dual energy X-ray absorptiometry (DXA) scanning (Prodigy Advance, Lunar Corporation, Madison, Wisconsin, USA). Also, bone mass measures is determined. Scanning was performed between 7 and 10 a.m. under standardized conditions after an overnight fast. All DXA scans are performed by the same experienced observer and the DXA software regional cut-points are visually inspected and manually adjusted if necessary. Body height and body weight are measured on a standard scale with subjects wearing light clothes.

NOTE: Helge EW, Andersen TR, Schmidt JF, Jørgensen NR, Hornstrup T, Krustrup P, Bangsbo J. Recreational football improves bone mineral density and bone turnover marker profile in elderly men. Scand J Med Sci Sports. 2014 Aug;24 Suppl 1:98-104. doi: 10.1111/sms.12239. Epub 2014 Jun 5.

Oral glucose tolerance test - An OGTT is performed after 0, 12 and 52 wks of the intervention period under standardized conditions. A glucose bolus of 75 g was ingested in 5 min according to WHO guidelines. Blood samples were collected from an antecubital vein immediately before and again after 15, 30, 60 and 120 min, and subsequently analyzed for glucose concentration.

NOTE: Testing session were performed at 0, 4 and 12 months.

Peripheral arterial tonometry (PAT): Measurements of the reactive hyperemic index (RHI) and the augmentation index (AI) are performed. In brief, a pneumatic probe was placed on the tip of each index finger and connected to a plethysmographic device (EndoPat-2000; Itamar Medical Ltd., Caesarea, Israel). After a 15-min resting period, baseline measurements were performed and then forearm ischemia was applied for 5 min through inflation of a BP cuff. After cuff deflation measurements are performed during reactive hyperemia, RHI (a measure of training, aging, and cardiovascular function and microvascular endothelial function) and AI (a measure of arterial stiffness) is determined by a computerized algorithm.

NOTE: Testing session were performed at 0, 4 and 12 months. Published in Schmidt JF[1], Andersen TR, Andersen LJ, Randers MB, Hornstrup T, Hansen PR, Bangsbo J, Krustrup P.Cardiovascular function is better in veteran football players than age-matched untrained elderly healthy men. Scand J Med Sci Sports. 2015 Feb;25(1):61-9. doi: 10.1111/sms.12153. Epub 2013 Dec 4. and in Schmidt JF, Hansen PR, Andersen TR, Andersen LJ, Hornstrup T, Krustrup P, Bangsbo J. Cardiovascular

adaptations to 4 and 12 months of football or strength training in 65- to 75-year-old untrained men. Scand J Med Sci Sports. 2014 Aug;24 Suppl 1:86-97. doi: 10.1111/sms.12217. Epub 2014 Jun 5.

Ekkokardiografi - At baseline and after 3 and 12 months, comprehensive transthoracic echocardiography is performed on a GE Vivid9 ultrasound machine with a 2.5 MHz transducer (GE Healthcare, Horten, Norway) in all subjects. The examination was performed with the subjects resting in a lateral decubitus position in a dark room by three experienced echocardiographers. All examinations is analyzed offline by an experienced echocardiographer in random order using the EchoPac software versionBT 12.0 (EchoPAC; GE Healthcare; Horten, Norway). All subjects were examined using the same protocol.

NOTE: Testing session were performed at 0, 4 and 12 months. Published in Schmidt JF[1], Andersen TR, Andersen LJ, Randers MB, Hornstrup T, Hansen PR, Bangsbo J, Krustrup P.Cardiovascular function is better in veteran football players than age-matched untrained elderly healthy men. Scand J Med Sci Sports. 2015 Feb;25(1):61-9. doi: 10.1111/sms.12153. Epub 2013 Dec 4.

Work test - A standardized exercise protocol consisting of 4-min of treadmill walking (4.5 km/h) and 4 min of jogging (7.0 km/h) separated by a 2-min period of passive rest, followedby 2-min of passive rest and finally an incremental cycling test to exhaustion is performed at each training session. During the incremental cycling test, subjects starts exercising at a work pace and load of 80 rpm and 40 W, respectively, after which the work load is increased by 20 W every 2-min until volitional fatigue. Pulmonary gas exchange (OxyconPro; VIASYS Healthcare, Hoechberg, Germany) and heart rate (HR; Polar Team System, Polar Electro Oy, Kempele, Finland) are measured continuously throughout the exercise protocol. VO2max is determined as the highest value achieved during a 30-s period, and the time to exhaustion (TTE) during the incremental test is noted. The individual maximal heart rate (HRmax) is determined as the highest value measured within a 15-s period during the test, and respiratory exchange ratio is calculated. .TheVO2max test was considered acceptable when respiratory exchange ratio (RER) > 1.05 and when a plateau in VO2 was observed.

NOTE: Partly published in Andersen TR[1], Schmidt JF, Nielsen JJ, Randers MB, Sundstrup E, Jakobsen MD, Andersen LL, Suetta C, Aagaard P, Bangsbo J, Krustrup P. Effect of football or

strength training on functional ability and physical performance in untrained old men. Scand J Med Sci Sports. 2014 Aug;24 Suppl 1:76-85. doi: 10.1111/sms.12245. Epub 2014 Jun 5.

Muscle function - Postural balance, maximal jumping height, maximal powerdevelopment og strength of the leg musculature is measured using an isokinetic dynamometer (KinCom). Postural balance is measured on a 5 cm balance beam. Also, at standardized sit-to-stand test and a sudden trunck loading test is performed.

NOTE: Partly published in Andersen TR[1], Schmidt JF, Nielsen JJ, Randers MB, Sundstrup E, Jakobsen MD, Andersen LL, Suetta C, Aagaard P, Bangsbo J, Krustrup P. Effect of football or strength training on functional ability and physical performance in untrained old men. Scand J Med Sci Sports. 2014 Aug;24 Suppl 1:76-85. doi: 10.1111/sms.12245. Epub 2014 Jun 5. An additional publication is currently being reweived.

Sprint ability and intermittent work capacity – After a standardized warm-up, two attempts at a 20-m sprinting distance are performed. On separate occasions, the subjects carry out a Yo-Yo IE1 test . Briefly, the Yo-Yo IE1 test consists of repeated 2 × 20 m runs at a progressively increased speed controlled by audio bleeps from a pre-recorded source.

NOTE: Sprint testing session were excluded from the test protocol at 0, 4 and 12 months due to safety precautions. Partly published in Andersen TR[1], Schmidt JF, Nielsen JJ, Randers MB, Sundstrup E, Jakobsen MD, Andersen LL, Suetta C, Aagaard P, Bangsbo J, Krustrup P. Effect of football or strength training on functional ability and physical performance in untrained old men. Scand J Med Sci Sports. 2014 Aug;24 Suppl 1:76-85. doi: 10.1111/sms.12245. Epub 2014 Jun 5.
