# Supplementary material for: The Effects of 52 Weeks of Soccer or Resistance Training on Body Composition and Muscle Function in +65-Year-Old Healthy Males – A Randomized Controlled Trial
Source: PLoS One. 2016 Feb 17;11(2):e0148236. doi: 10.1371/journal.pone.0148236 (PMC4757560; doi:10.1371/journal.pone.0148236)
Supplement: S1 Table — (PDF) [file pone.0148236.s004.pdf]

		Total Body Mass (BM) (kg)				
0 weeks values	SG 1	69,50	RG 1	59,30	CG 1	79,20
	SG 2	72,30	RG 2	74,60	CG 2	105,70
	SG 3	80,30	RG 3	89,70	CG 3	81,60
	SG 4	71,80	RG 4	88,10	CG 4	108,90
	SG 5	80,70	RG 5	90,50	CG 5	74,40
	SG 6	83,30	RG 6	87,60	CG 6	83,70
	SG 7	92,80	RG 7	92,90	CG 7	88,00
	SG 8	85,80	RG 8	89,00	CG 8	93,00
	SG 9	62,30	RG 9	100,70		
16 weeks values	SG 1	68,00	RG 1	60,00	CG 1	78,00
	SG 2	72,30	RG 2	74,30	CG 2	105,20
	SG 3	77,00	RG 3	90,60	CG 3	81,90
	SG 4	72,70	RG 4	88,20	CG 4	105,10
	SG 5	79,40	RG 5	87,70	CG 5	74,50
	SG 6	81,80	RG 6	86,00	CG 6	81,60
	SG 7	91,40	RG 7	96,40	CG 7	89,90
	SG 8	85,10	RG 8	88,20	CG 8	93,50
	SG 9	60,40	RG 9	96,80		
52 weeks values	SG 1	65,20	RG 1	58,70	CG 1	79,00
	SG 2	69,60	RG 2	76,10	CG 2	107,10
	SG 3	74,00	RG 3	90,60	CG 3	85,10
	SG 4	72,00	RG 4	86,90	CG 4	106,70
	SG 5	81,50	RG 5	88,30	CG 5	76,90
	SG 6	81,80	RG 6	86,30	CG 6	84,30
	SG 7	89,40	RG 7	95,90	CG 7	89,90
	SG 8	82,70	RG 8	86,40	CG 8	93,50
	SG 9	61,30	RG 9	100,50		

		Body Mass Index (BMI)				
0 weeks values	SG 1	25,53	RG 1	24,37	CG 1	22,89
	SG 2	26,56	RG 2	23,55	CG 2	34,51
	SG 3	28,79	RG 3	31,04	CG 3	27,58
	SG 4	23,99	RG 4	28,44	CG 4	35,56
	SG 5	24,36	RG 5	27,62	CG 5	25,15
	SG 6	28,49	RG 6	24,01	CG 6	25,55
	SG 7	28,96	RG 7	30,33	CG 7	25,71
	SG 8	29,34	RG 8	28,09	CG 8	26,88
	SG 9	17,82	RG 9	29,42		
16 weeks values	SG 1	24,98	RG 1	24,65	CG 1	22,55
	SG 2	26,56	RG 2	23,45	CG 2	34,35
	SG 3	27,61	RG 3	31,35	CG 3	27,68
	SG 4	24,29	RG 4	28,47	CG 4	34,32
	SG 5	23,97	RG 5	26,77	CG 5	25,18
	SG 6	27,97	RG 6	23,57	CG 6	24,91
	SG 7	28,53	RG 7	31,48	CG 7	26,27
	SG 8	29,10	RG 8	27,84	CG 8	27,03
	SG 9	17,27	RG 9	28,28		
52 weeks values	SG 1	23,95	RG 1	24,12	CG 1	22,84
	SG 2	25,56	RG 2	24,02	CG 2	34,97
	SG 3	26,53	RG 3	31,35	CG 3	28,77
	SG 4	24,06	RG 4	28,05	CG 4	34,84
	SG 5	24,60	RG 5	26,95	CG 5	25,99
	SG 6	27,97	RG 6	23,66	CG 6	25,73
	SG 7	27,90	RG 7	31,31	CG 7	26,27
	SG 8	28,28	RG 8	27,27	CG 8	27,03
	SG 9	17,53	RG 9	29,36		

		SOD-2 expression (log(value-to-group PRE mean) (A.U.))				
0 weeks values	SG 1	-0,10	RG 1	-0,11	CG 1	0,00
	SG 2		RG 2	-0,23	CG 2	0,09
	SG 3	0,05	RG 3	-0,09	CG 3	-0,49
	SG 4	-0,35	RG 4	0,28	CG 4	-0,40
	SG 5	0,08	RG 5	-0,39	CG 5	0,46
	SG 6	-0,26	RG 6	-0,06	CG 6	0,05
	SG 7	-0,17	RG 7	-0,08	CG 7	-0,16
	SG 8	-0,29	RG 8	0,37	CG 8	-0,50
	SG 9	0,43	RG 9	-0,38		
16 weeks values	SG 1	0,11	RG 1	-0,16	CG 1	0,22
	SG 2		RG 2	-0,15	CG 2	0,03
	SG 3	0,26	RG 3	-0,13	CG 3	0,00
	SG 4	-0,09	RG 4	0,25	CG 4	-0,74
	SG 5	0,43	RG 5	-0,26	CG 5	0,38
	SG 6	-0,24	RG 6	0,19	CG 6	-0,10
	SG 7	-0,17	RG 7	0,14	CG 7	-0,28
	SG 8	0,05	RG 8	0,19	CG 8	-0,34
	SG 9	-0,02	RG 9	-0,28		
52 weeks values	SG 1	0,18	RG 1	0,04	CG 1	-0,04
	SG 2		RG 2	-0,25	CG 2	0,05
	SG 3	0,24	RG 3	-0,13	CG 3	-0,03
	SG 4	0,12	RG 4	0,14	CG 4	-0,74
	SG 5	0,40	RG 5	-0,24	CG 5	0,08
	SG 6	-0,02	RG 6	0,10	CG 6	0,01
	SG 7	0,17	RG 7	0,26	CG 7	-0,28
	SG 8	0,15	RG 8	0,22	CG 8	-0,34
	SG 9	0,01	RG 9	-0,19		

		Akt-2 expression (log(value-to-group PRE mean) (A.U.))				
0 weeks values	SG 1	0,03	RG 1	-0,02	CG 1	-0,08
	SG 2		RG 2	-0,09	CG 2	0,06
	SG 3	-0,13	RG 3	0,15	CG 3	0,06
	SG 4	0,13	RG 4	-0,10	CG 4	-0,08
	SG 5	-0,03	RG 5	-0,11	CG 5	0,02
	SG 6	-0,02	RG 6	-0,05	CG 6	-0,08
	SG 7	0,02	RG 7	-0,01	CG 7	0,00
	SG 8	-0,07	RG 8	0,09	CG 8	0,06
	SG 9	0,03	RG 9	0,06		
16 weeks values	SG 1	0,27	RG 1	0,08	CG 1	0,04
	SG 2		RG 2	-0,13	CG 2	-0,03
	SG 3	-0,01	RG 3	0,10	CG 3	0,08
	SG 4	0,15	RG 4	0,10	CG 4	-0,11
	SG 5	0,12	RG 5	0,01	CG 5	0,03
	SG 6	-0,24	RG 6	0,15	CG 6	0,18
	SG 7	0,02	RG 7	0,02	CG 7	-0,01
	SG 8	-0,16	RG 8	0,16	CG 8	0,07
	SG 9	0,02	RG 9	0,15		
52 weeks values	SG 1	0,04	RG 1	0,09	CG 1	-0,07
	SG 2		RG 2	-0,03	CG 2	0,05
	SG 3	-0,18	RG 3	0,10	CG 3	0,00
	SG 4	0,45	RG 4	0,03	CG 4	-0,16
	SG 5	-0,05	RG 5	0,13	CG 5	0,11
	SG 6	-0,03	RG 6	0,06	CG 6	-0,05
	SG 7	0,00	RG 7	0,17	CG 7	-0,01
	SG 8	-0,07	RG 8	0,10	CG 8	0,07
	SG 9	0,05	RG 9	0,24		

	Total Lean Body Mass (LBM) (kg)						Upper Body Lean Mass (kg)					
0 weeks values	SG 1	51,41	RG 1	49,56	CG 1	58,43	SG 1	31,23	RG 1	30,10	CG 1	34,20
	SG 2	51,95	RG 2	47,89	CG 2	62,65	SG 2	30,39	RG 2	27,61	CG 2	39,73
	SG 3	53,13	RG 3	54,23	CG 3	54,24	SG 3	31,68	RG 3	30,50	CG 3	31,56
	SG 4	51,58	RG 4	57,09	CG 4	66,94	SG 4	32,11	RG 4	32,67	CG 4	40,59
	SG 5	57,02	RG 5	58,29	CG 5	50,33	SG 5	34,04	RG 5	32,69	CG 5	30,20
	SG 6	52,39	RG 6	58,49	CG 6	59,09	SG 6	30,86	RG 6	33,88	CG 6	34,56
	SG 7	59,49	RG 7	57,59	CG 7	59,73	SG 7	35,10	RG 7	34,87	CG 7	34,02
	SG 8	49,55	RG 8	60,36	CG 8	61,66	SG 8	29,71	RG 8	33,77	CG 8	37,19
	SG 9	50,48	RG 9	64,55			SG 9	30,17	RG 9	36,51		
16 weeks values	SG 1	51,50	RG 1	49,20	CG 1	57,59	SG 1	30,56	RG 1	29,85	CG 1	34,20
	SG 2	51,95	RG 2	50,85	CG 2	63,97	SG 2	30,39	RG 2	29,33	CG 2	41,82
	SG 3	54,40	RG 3	54,17	CG 3	55,30	SG 3	32,50	RG 3	30,31	CG 3	32,26
	SG 4	52,99	RG 4	59,47	CG 4	63,18	SG 4	31,96	RG 4	34,91	CG 4	38,74
	SG 5	56,75	RG 5	58,96	CG 5	50,75	SG 5	32,99	RG 5	33,24	CG 5	30,17
	SG 6	51,28	RG 6	59,90	CG 6	58,36	SG 6	29,68	RG 6	34,82	CG 6	34,24
	SG 7	60,07	RG 7	60,04	CG 7	60,15	SG 7	35,06	RG 7	36,63	CG 7	34,32
	SG 8	47,74	RG 8	60,66	CG 8	63,52	SG 8	27,89	RG 8	34,33	CG 8	38,14
	SG 9	49,92	RG 9	65,04			SG 9	29,55	RG 9	37,55		
52 weeks values	SG 1	50,61	RG 1	48,46	CG 1	58,26	SG 1	30,06	RG 1	28,87	CG 1	34,66
	SG 2	50,61	RG 2	50,27	CG 2	67,00	SG 2	29,95	RG 2	29,11	CG 2	44,49
	SG 3	54,79	RG 3	54,17	CG 3	56,99	SG 3	33,46	RG 3	30,31	CG 3	32,88
	SG 4	50,65	RG 4	59,27	CG 4	64,43	SG 4	30,76	RG 4	34,18	CG 4	38,27
	SG 5	55,79	RG 5	57,64	CG 5	51,53	SG 5	32,38	RG 5	33,16	CG 5	30,41
	SG 6	51,90	RG 6	59,50	CG 6	59,49	SG 6	30,65	RG 6	34,63	CG 6	34,36
	SG 7	58,11	RG 7	60,73	CG 7	60,15	SG 7	32,78	RG 7	36,39	CG 7	34,32
	SG 8	48,75	RG 8	61,33	CG 8	63,52	SG 8	29,35	RG 8	34,75	CG 8	38,14
	SG 9	50,44	RG 9	64,55			SG 9	30,37	RG 9	36,51		

	Glut-4 expression (log(value-to-group PRE mean) (A.U.))						Follistatin expression (log(value-to-group PRE mean) (A.U.))					
0 weeks values	SG 1	-0,32	RG 1	0,42	CG 1	0,33	SG 1	-0,23	RG 1	-0,65	CG 1	0,65
	SG 2		RG 2	-0,28	CG 2	-0,40	SG 2		RG 2	-0,28	CG 2	0,02
	SG 3	0,01	RG 3	-0,03	CG 3	-0,08	SG 3	0,33	RG 3	-0,48	CG 3	-0,31
	SG 4	0,15	RG 4	-0,03	CG 4	-0,30	SG 4	-0,07	RG 4	-0,24	CG 4	-0,54
	SG 5	-0,26	RG 5	-0,54	CG 5	-0,10	SG 5	-0,51	RG 5	0,39	CG 5	-0,08
	SG 6	0,46	RG 6	-0,13	CG 6	-0,12	SG 6	-0,40	RG 6	0,31	CG 6	-0,44
	SG 7	-0,22	RG 7	0,07	CG 7	0,37	SG 7	0,08	RG 7	0,25	CG 7	-0,97
	SG 8	-0,44	RG 8	0,09	CG 8	-0,57	SG 8	-0,33	RG 8	-0,12	CG 8	-0,43
	SG 9	-0,16	RG 9	-0,26			SG 9	0,31	RG 9	-0,52		
16 weeks values	SG 1	0,16	RG 1	0,48	CG 1	-0,03	SG 1	-0,30	RG 1	-0,30	CG 1	0,00
	SG 2		RG 2	-0,22	CG 2	-0,34	SG 2		RG 2	-0,07	CG 2	-0,11
	SG 3	-0,16	RG 3	-0,04	CG 3	-0,14	SG 3	0,28	RG 3	-0,29	CG 3	-0,30
	SG 4	0,17	RG 4	-0,12	CG 4	-0,40	SG 4	0,07	RG 4	-0,05	CG 4	-0,51
	SG 5	-0,35	RG 5	-0,61	CG 5	-0,02	SG 5	-0,33	RG 5	-0,02	CG 5	0,12
	SG 6	0,34	RG 6	-0,12	CG 6	-0,09	SG 6	-0,49	RG 6	0,21	CG 6	-0,27
	SG 7	-0,22	RG 7	0,06	CG 7	0,34	SG 7	0,08	RG 7	0,31	CG 7	-0,89
	SG 8	-0,27	RG 8	0,11	CG 8	-0,45	SG 8	-0,12	RG 8	0,06	CG 8	-0,81
	SG 9	-0,15	RG 9	-0,29			SG 9	0,32	RG 9	-0,26		
52 weeks values	SG 1	0,09	RG 1	0,43	CG 1	0,10	SG 1	-0,59	RG 1	-0,68	CG 1	0,39
	SG 2		RG 2	-0,47	CG 2	-0,60	SG 2		RG 2	-0,29	CG 2	-0,06
	SG 3	0,00	RG 3	-0,04	CG 3	0,19	SG 3	0,17	RG 3	-0,29	CG 3	-0,35
	SG 4	0,24	RG 4	-0,15	CG 4	-0,48	SG 4	-0,28	RG 4	-0,29	CG 4	-0,23
	SG 5	-0,14	RG 5	-0,67	CG 5	-0,10	SG 5	-0,10	RG 5	-0,21	CG 5	0,09
	SG 6	0,58	RG 6	-0,23	CG 6	-0,07	SG 6	-0,94	RG 6	0,14	CG 6	-0,23
	SG 7	0,04	RG 7	0,02	CG 7	0,34	SG 7	0,14	RG 7	-0,47	CG 7	-0,89
	SG 8	-0,35	RG 8	0,02	CG 8	-0,45	SG 8	-0,24	RG 8	0,03	CG 8	-0,81
	SG 9	-0,12	RG 9	-0,38			SG 9	0,07	RG 9	-0,45		

	Leg lean mass (kg)						A/G-ratio					
0 weeks values	SG 1	16,50	RG 1	15,73	CG 1	19,96	SG 1	1,41	RG 1	0,60	CG 1	1,44
	SG 2	17,76	RG 2	16,76	CG 2	19,05	SG 2	1,10	RG 2	0,98	CG 2	1,46
	SG 3	17,64	RG 3	19,28	CG 3	18,92	SG 3	1,61	RG 3	1,33	CG 3	1,62
	SG 4	16,00	RG 4	20,43	CG 4	22,41	SG 4	1,12	RG 4	1,50	CG 4	1,47
	SG 5	19,99	RG 5	21,76	CG 5	16,35	SG 5	1,01	RG 5	1,48	CG 5	1,42
	SG 6	17,62	RG 6	21,05	CG 6	20,52	SG 6	1,27	RG 6	1,22	CG 6	1,41
	SG 7	20,64	RG 7	18,67	CG 7	21,54	SG 7	1,09	RG 7	1,16	CG 7	1,40
	SG 8	16,28	RG 8	22,42	CG 8	20,06	SG 8	1,23	RG 8	1,56	CG 8	0,97
	SG 9	16,84	RG 9	23,71			SG 9	0,98	RG 9	1,64		
16 weeks values	SG 1	17,135	RG 1	15,53	CG 1	19,38	SG 1	1,50	RG 1	0,82	CG 1	1,47
	SG 2	17,761	RG 2	17,90	CG 2	18,43	SG 2	1,10	RG 2	1,03	CG 2	1,45
	SG 3	18,034	RG 3	19,61	CG 3	19,26	SG 3	1,49	RG 3	1,45	CG 3	1,56
	SG 4	17,193	RG 4	20,79	CG 4	20,13	SG 4	1,13	RG 4	1,56	CG 4	1,36
	SG 5	20,427	RG 5	21,91	CG 5	16,54	SG 5	0,89	RG 5	1,43	CG 5	1,47
	SG 6	17,747	RG 6	21,47	CG 6	20,19	SG 6	1,33	RG 6	1,31	CG 6	1,44
	SG 7	21,306	RG 7	19,51	CG 7	21,23	SG 7	1,10	RG 7	1,11	CG 7	1,30
	SG 8	16,197	RG 8	22,01	CG 8	20,88	SG 8	1,22	RG 8	1,42	CG 8	1,05
	SG 9	16,874	RG 9	23,89			SG 9	1,19	RG 9	1,63		
52 weeks values	SG 1	16,48	RG 1	15,91	CG 1	19,69	SG 1	1,29	RG 1	0,67	CG 1	1,50
	SG 2	16,78	RG 2	16,92	CG 2	18,53	SG 2	1,12	RG 2	0,91	CG 2	1,52
	SG 3	17,62	RG 3	19,61	CG 3	20,55	SG 3	1,43	RG 3	1,45	CG 3	1,70
	SG 4	16,23	RG 4	21,27	CG 4	21,95	SG 4	1,06	RG 4	1,49	CG 4	1,49
	SG 5	20,08	RG 5	20,68	CG 5	17,14	SG 5	1,00	RG 5	1,34	CG 5	1,51
	SG 6	17,28	RG 6	20,57	CG 6	21,18	SG 6	1,21	RG 6	1,23	CG 6	1,49
	SG 7	20,94	RG 7	20,42	CG 7	21,23	SG 7	1,10	RG 7	1,18	CG 7	1,30
	SG 8	15,76	RG 8	22,34	CG 8	20,88	SG 8	1,15	RG 8	1,46	CG 8	1,05
	SG 9	16,54	RG 9	24,48			SG 9	1,01	RG 9	1,65		

	OGTT Glucose AUC (A.U.)						TC/HDL-cholesterol ratio					
0 weeks values	SG 1	15,20	RG 1	12,68	CG 1	17,63	SG 1	4,24	RG 1	2,60	CG 1	3,44
	SG 2	14,30	RG 2	17,70	CG 2	16,36	SG 2	3,80	RG 2	4,20	CG 2	4,36
	SG 3	15,04	RG 3	15,46	CG 3	15,86	SG 3	3,31	RG 3	3,83	CG 3	4,13
	SG 4	18,55	RG 4	16,33	CG 4	17,90	SG 4	3,20	RG 4	3,44	CG 4	4,38
	SG 5	11,66	RG 5	14,58	CG 5	17,15	SG 5	2,93	RG 5	3,50	CG 5	4,15
	SG 6	25,15	RG 6	13,83	CG 6	14,31	SG 6	5,45	RG 6	3,14	CG 6	2,53
	SG 7	16,45	RG 7	16,95	CG 7	14,11	SG 7	4,00	RG 7	3,65	CG 7	3,07
	SG 8		RG 8	13,33	CG 8	13,43	SG 8		RG 8	4,64	CG 8	2,56
	SG 9	14,58	RG 9	12,51			SG 9	2,81	RG 9	4,42		
16 weeks values	SG 1	16,10	RG 1	11,80	CG 1	14,68	SG 1	3,53	RG 1	2,48	CG 1	3,00
	SG 2	11,80	RG 2	18,18	CG 2	16,48	SG 2	3,47	RG 2	3,81	CG 2	4,42
	SG 3	12,43	RG 3	14,19	CG 3	18,19	SG 3	3,07	RG 3	3,83	CG 3	3,71
	SG 4	17,33	RG 4	14,21	CG 4	19,79	SG 4	2,94	RG 4	3,73	CG 4	4,08
	SG 5	9,51	RG 5	15,64	CG 5	19,30	SG 5	3,07	RG 5	3,25	CG 5	3,53
	SG 6	21,88	RG 6	14,39	CG 6	13,98	SG 6	4,77	RG 6	2,87	CG 6	2,69
	SG 7	11,01	RG 7	20,19	CG 7	11,68	SG 7	3,79	RG 7	3,24	CG 7	3,05
	SG 8		RG 8	12,18	CG 8	15,45	SG 8		RG 8	4,33	CG 8	2,23
	SG 9	13,34	RG 9	14,79			SG 9	2,48	RG 9	4,08		
52 weeks values	SG 1	13,59	RG 1	16,51	CG 1	16,88	SG 1	3,23	RG 1	2,34	CG 1	3,39
	SG 2	14,38	RG 2	15,01	CG 2	16,26	SG 2	3,55	RG 2	3,64	CG 2	4,55
	SG 3	14,78	RG 3	17,55	CG 3	15,15	SG 3	2,51	RG 3	3,83	CG 3	4,33
	SG 4	19,00	RG 4	14,35	CG 4	19,85	SG 4	2,51	RG 4	3,25	CG 4	4,61
	SG 5	11,59	RG 5	14,79	CG 5	17,79	SG 5	2,98	RG 5	3,27	CG 5	3,78
	SG 6	23,03	RG 6	15,41	CG 6	14,01	SG 6	4,59	RG 6	2,67	CG 6	2,41
	SG 7	18,00	RG 7	16,94	CG 7	11,68	SG 7	3,89	RG 7	2,64	CG 7	3,05
	SG 8		RG 8	13,53	CG 8	15,45	SG 8		RG 8	4,02	CG 8	2,23
	SG 9	13,36	RG 9	13,80			SG 9	2,68	RG 9	3,58		

Maximal PFK enzyme activity

0 weeks values	SG 1	326,90	RG 1	232,690903	CG 1	268,441742
	SG 2		RG 2	283,715894	CG 2	294,121284
	SG 3	260,58	RG 3	369,540927	CG 3	282,825627
	SG 4	206,48	RG 4	225,425422	CG 4	300,157472
	SG 5	249,70	RG 5	120,9551	CG 5	197,61811
	SG 6	226,43	RG 6	200,039564	CG 6	311,284002
	SG 7	280,45	RG 7	271,47767	CG 7	178,620579
	SG 8	223,68	RG 8	144,760544	CG 8	227,435225
	SG 9	231,57	RG 9	268,607354		
16 weeks values	SG 1	390,32	RG 1	310,53	CG 1	340,44
	SG 2		RG 2	283,72	CG 2	157,26
	SG 3	268,18	RG 3	296,58	CG 3	265,34
	SG 4	242,00	RG 4	199,59	CG 4	292,01
	SG 5	313,53	RG 5	254,84	CG 5	169,00
	SG 6	229,99	RG 6	252,23	CG 6	340,56
	SG 7	260,13	RG 7	311,26	CG 7	166,64
	SG 8	226,88	RG 8	205,38	CG 8	266,51
	SG 9	279,85	RG 9	324,84		
52 weeks values	SG 1	307,66	RG 1	287,88	CG 1	257,67
	SG 2		RG 2	300,60	CG 2	201,79
	SG 3	248,10	RG 3	296,58	CG 3	288,74
	SG 4	228,49	RG 4	235,86	CG 4	268,23
	SG 5	279,19	RG 5	205,59	CG 5	225,23
	SG 6	237,55	RG 6	204,80	CG 6	255,44
	SG 7	269,48	RG 7	323,64	CG 7	166,64
	SG 8	249,74	RG 8	129,85	CG 8	266,51
	SG 9	246,59	RG 9	288,06		

Maximal CS enzyme activity

0 weeks values	SG 1	19,09	RG 1	28,78	CG 1	23,40
	SG 2		RG 2	23,48	CG 2	22,65
	SG 3	23,25	RG 3	32,80	CG 3	25,01
	SG 4	25,14	RG 4	27,46	CG 4	20,24
	SG 5	15,15	RG 5	17,77	CG 5	22,97
	SG 6	15,49	RG 6	25,24	CG 6	27,20
	SG 7	15,04	RG 7	24,80	CG 7	20,62
	SG 8	18,08	RG 8	23,81	CG 8	13,94
	SG 9	24,92	RG 9	24,47		
16 weeks values	SG 1	26,90	RG 1	24,14	CG 1	32,59
	SG 2		RG 2	23,48	CG 2	21,00
	SG 3	34,50	RG 3	35,99	CG 3	31,16
	SG 4	31,04	RG 4	31,52	CG 4	13,44
	SG 5	25,36	RG 5	28,93	CG 5	24,34
	SG 6	14,85	RG 6	35,00	CG 6	24,69
	SG 7	20,24	RG 7	25,23	CG 7	26,07
	SG 8	19,51	RG 8	29,43	CG 8	17,58
	SG 9	26,35	RG 9	25,95		
52 weeks values	SG 1	26,31	RG 1	27,89	CG 1	32,75
	SG 2		RG 2	16,15	CG 2	19,40
	SG 3	36,94	RG 3	35,99	CG 3	34,83
	SG 4	29,93	RG 4	26,90	CG 4	15,08
	SG 5	23,74	RG 5	22,70	CG 5	20,71
	SG 6	23,18	RG 6	32,71	CG 6	23,76
	SG 7	26,87	RG 7	26,30	CG 7	26,07
	SG 8	23,34	RG 8	22,35	CG 8	17,58
	SG 9	24,80	RG 9	24,28		

Maximal HAD enzyme activity

0 weeks values	SG 1	14,59	RG 1	16,43	CG 1	16,57
	SG 2		RG 2	19,37	CG 2	16,33
	SG 3	13,64	RG 3	22,54	CG 3	18,39
	SG 4	15,77	RG 4	23,50	CG 4	15,05
	SG 5	15,97	RG 5	17,56	CG 5	15,79
	SG 6	16,77	RG 6	20,35	CG 6	15,92
	SG 7	17,08	RG 7	18,47	CG 7	20,94
	SG 8	18,59	RG 8	17,59	CG 8	15,56
	SG 9	22,42	RG 9	16,87		
16 weeks values	SG 1	12,90	RG 1	15,91	CG 1	20,38
	SG 2		RG 2	19,37	CG 2	15,22
	SG 3	19,65	RG 3	22,78	CG 3	19,45
	SG 4	15,64	RG 4	23,14	CG 4	13,67
	SG 5	22,22	RG 5	20,99	CG 5	15,44
	SG 6	14,96	RG 6	22,20	CG 6	12,89
	SG 7	17,94	RG 7	19,12	CG 7	24,62
	SG 8	16,47	RG 8	16,73	CG 8	21,35
	SG 9	23,86	RG 9	15,26		
52 weeks values	SG 1	17,47	RG 1	15,77	CG 1	20,69
	SG 2		RG 2	11,48	CG 2	13,39
	SG 3	22,37	RG 3	22,78	CG 3	22,64
	SG 4	13,65	RG 4	22,82	CG 4	12,74
	SG 5	19,53	RG 5	17,77	CG 5	15,43
	SG 6	20,40	RG 6	20,94	CG 6	18,68
	SG 7	18,56	RG 7	19,98	CG 7	24,62
	SG 8	17,29	RG 8	16,18	CG 8	21,35
	SG 9	20,10	RG 9	15,07		
